# Recent Advances in Reactive Microdroplets for Clean Water and Energy

**DOI:** 10.1002/adma.202509486

**Published:** 2025-07-30

**Authors:** Qiuyun Lu, Boubakar Sanogo, Tanay Kumar, Ben Bin Xu, Xuehua Zhang

**Affiliations:** ^1^ Department of Chemical and Materials Engineering University of Alberta Edmonton Alberta T6G 1H9 Canada; ^2^ Mechanical and Construction Engineering, Faculty of Engineering and Environment Northumbria University Newcastle Upon Tyne NE1 8ST UK; ^3^ Physics of Fluids Group and Max Planck Center for Complex Fluid Dynamics University of Twente 7500 AE Enschede The Netherlands

**Keywords:** clean energy, functional materials, materials synthesis, microdroplets, microreactors, water remediation

## Abstract

Microdroplets have emerged as powerful and sustainable platforms for the design and synthesis of functional materials under mild and environmentally friendly conditions. Their unique physicochemical environments ‐ characterized by high surface‐to‐volume ratios and confined internal space ‐ enable precise control over mass and heat transfer, interfacial energy conversion, and chemical reactions. These features have been harnessed in two main ways: first, by employing microdroplets as microreactors for the fabrication of advanced materials such as polymeric microlenses, artificial compound eyes, metal oxide nanoparticles, and metal‐organic framework microstructures; and second, by using microdroplets as reactive entities to accelerate interfacial reactions relevant to hydrogen and biodiesel production, as well as nitrogen and carbon dioxide fixation. Together, these strategies have driven significant advances in clean energy generation, environmental monitoring, and water treatment. This review provides a critical overview of recent progress in microdroplet‐assisted synthesis of functional materials and their integration in energy and environmental technologies. An emerging direction in the integration of microdroplet‐based systems into adaptive sensing and human‐machine interfaces driven by artificial intelligence is also highlighted.

## Graphical abstract

Reactive microdroplets enable precise and sustainable chemistry at small scales. This review explores their role as confined reactors and dynamic interfaces for synthesizing functional materials, fuels, and microdevices. It offers a critical perspective on how droplet‐based platforms can drive next‐generation technologies in clean energy, environmental remediation, and smart sensing.

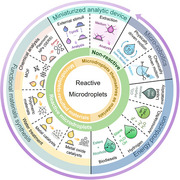

## 1 Introduction

The urgent global demand for more sustainable and cleaner strategies in functional material fabrication has driven the development of innovative methods that minimize waste, reduce energy consumption, and mitigate environmental impact. Among these, microdroplets serve as a cornerstone in the rational design and emerging functionalities of advanced materials. Their unique properties, due to their small size and high surface‐to‐volume ratio, affords opportunities for controlling over interfacial interactions and mass transfer,^[^ ^1^, ^2^, ^3^, ^4^, ^5^ ^]^ clean energy generation, and heat management.^[^ ^6^, ^7^ ^]^ A particularly compelling illustration of the impact of microdroplets is from the domains of biomimetic materials and interfacial science. In these fields, the seminal contributions of Lei Jiang and his co‐workers were groundbreaking, in pioneering the fabrication of functional surfaces toward controlled droplets behavior.^[^ ^8^, ^9^, ^10^, ^11^, ^12^, ^13^, ^14^ ^]^

Recently, an expanding body of research has focused on elucidating the characteristics of microdroplet reactions, aiming to uncover the fundamental mechanisms behind their unique reactivity.^[^ ^15^, ^16^, ^17^ ^]^ The scope of this research has been expanded toward harnessing microdroplets as sophisticated platforms for the rational design and fabrication of advanced functional materials. Owing to their confined volumes and interfacial properties, microdroplets offer precise spatial and temporal control over nucleation, growth, and self‐assembly processes.^[^ ^18^, ^19^, ^20^ ^]^ Their miniature scale also enhances reproducibility, manipulation accuracy, and compatibility with automated synthesis platforms.^[^ ^21^ ^]^ As a consequence, reactive microdroplets have been instrumental in the design of a broad range of functional materials. These include polymeric optic units,^[^ ^22^, ^23^, ^24^ ^]^ micro‐ and nanoparticles of metal oxides,^[^ ^25^, ^26^, ^27^, ^28^, ^29^, ^30^ ^]^ and metal–organic frameworks (MOF).^[^ ^31^, ^32^, ^33^ ^]^ Materials synthesized through these microdroplet‐based strategies have demonstrated superior performance in real‐time and ultra‐sensitive detection of otherwise difficult‐to‐identify contaminants.^[^ ^34^, ^35^, ^36^ ^]^ They have found significant utility in water and wastewater treatment as effective adsorbents,^[^ ^29^, ^31^ ^]^ filtration membrane components,^[^ ^30^, ^37^ ^]^ optical materials for enhanced light harvesting,^[^ ^38^, ^39^ ^]^ and reusable photocatalysts for pollutant degradation and separation.^[^ ^31^, ^40^ ^]^

In parallel, microdroplets have also been employed as functional micro‐compartments to drive a wide range of physical and chemical processes. By leveraging their confined reaction environments, microdroplets have enabled advances in both analytical and synthetic applications. For instance, they have been extensively applied in processing tasks such as chemical concentration,^[^ ^41^, ^42^ ^]^ molecular detection,^[^ ^43^, ^44^ ^]^ and reaction monitoring.^[^ ^45^, ^46^ ^]^ Furthermore, microdroplet‐based systems have demonstrated efficiency in producing clean and sustainable energy carriers,^[^ ^47^, ^48^, ^49^ ^]^ with recent studies highlighting their potential for nitrogen or carbon dioxide fixation and ammonia synthesis.^[^ ^50^, ^51^, ^52^ ^]^ They have also been shown to be effective in facilitating reactions between immiscible phases, including the dehydrogenation of liquid organic hydrogen carriers for hydrogen release,^[^ ^53^, ^54^ ^]^ and the transesterification of biomass‐derived lipids for biodiesel production.^[^ ^55^, ^56^ ^]^

**Figure 1:**
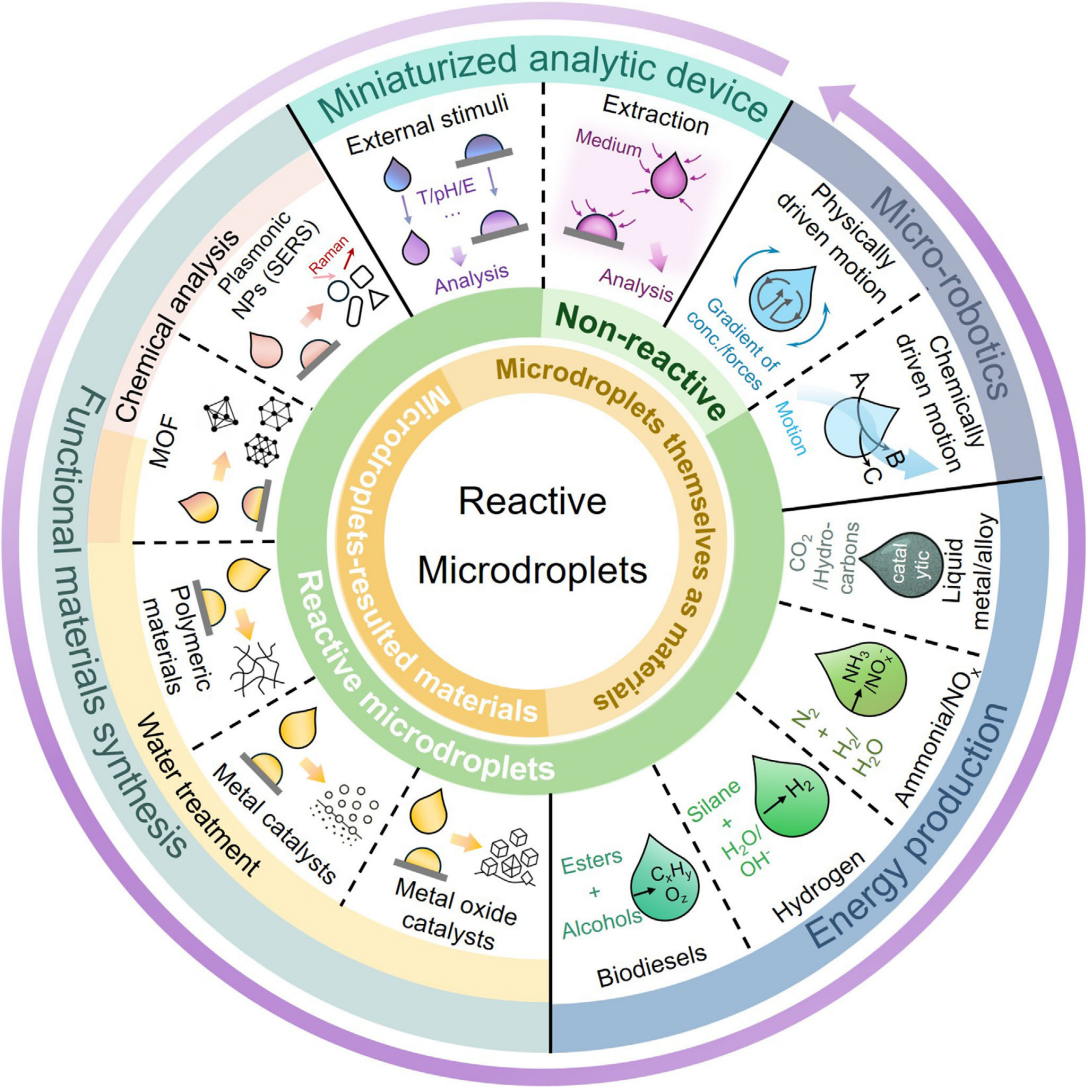
Overview of microdroplet‐based strategies for functional material synthesis, energy carrier production, self‐propelled microswimmers, and miniaturized analytical devices.

The application of microdroplets as both confined synthesis environments and functional micro‐compartments has significantly advanced the development of next‐generation functional materials. Despite these progresses, a comprehensive and dedicated review that systematically examines the integrated role of microdroplet‐based strategies in clean energy and environmental remediation is currently lacking. Therefore, a critical overview of recent progress in these domains is both timely and valuable to the advanced materials research community. In this review, we explore recent developments in leveraging microdroplets for the synthesis and deployment of functional materials aimed at clean energy production and water treatment (**Figure** **1**). Particular emphasis is placed on how droplet‐scale systems enable precise and programmable material synthesis and offer enhanced control over interfacial reaction kinetics. It is important to note that this review does not delve into the fundamental mechanisms underlying the physicochemical properties of microdroplets. Instead, Section 2 outlines the leading hypotheses and provides contextual insight into the ongoing debate on microdroplet‐enabled reaction acceleration.

In Section 3, we highlight recent advances in the use of reactive microdroplets for chemical sensing and analysis. The section begins by examining the intrinsic physicochemical properties of non‐reactive microdroplets that underpin their functionality, followed by a discussion of how these features are harnessed in reactive systems for analyte enrichment and detection. Building on this foundation, Section 4 highlights how microdroplet‐assisted strategies drive the synthesis of advanced materials for pollutant removal and water remediation. The review then transitions to confined interfacial environments, where microdroplets accelerate key chemical reactions, particularly those involved in the production of clean energy carriers. Next, we highlight recent progress in the design of self‐propelled microdroplets driven by external stimuli or Marangoni forces to achieve autonomous motion. Finally, Section 7 presents emerging directions, particularly how artificial intelligence (AI) is driving the rational design of advanced materials and shaping next‐generation technologies for clean water and energy through advanced AI‐human interfaces.

## 2 Microdroplets Reactivity: Competing Theories and Applications

A compelling body of experimental evidence has demonstrated the remarkable ability of microdroplets to accelerate chemical reactions.^[^ ^57^, ^58^, ^59^ ^]^ This acceleration spans diverse reaction classes, such as hydrolysis and condensation,^[^ ^3^, ^60^, ^61^ ^]^ redox processes,^[^ ^16^, ^62^, ^63^ ^]^ and intricate cascade catalytic sequences.^[^ ^64^, ^65^, ^66^ ^]^ Rate enhancements often reach several orders of magnitude compared to conventional bulk systems, frequently occurring without external stimuli like heating or agitation, and without added catalysts. Despite significant progress in both experimental and computational investigations, the fundamental mechanisms underlying this enhanced reactivity remain a subject of intense scientific debate. Several hypotheses have been proposed, including the influence of high surface‐to‐volume ratios, the presence of strong interfacial electric fields, elevated interfacial potential energy, and the formation of reactive redox intermediates at microdroplet interfaces.^[^ ^2^, ^67^ ^]^ These unique features are recognized as fundamental parameters for engineering functional materials, as well as for the production of clean energy carriers.

A common principle across the diverse applications surveyed in this review is the critical role of surface area, particularly the high surface‐to‐volume ratio inherent to microdroplets. This concept was rigorously investigated by Fallah‐Araghi et al.^[^ ^68^ ^]^ who demonstrated an inverse correlation between droplet radius and both the apparent equilibrium constant and forward reaction rate. Their seminal work also revealed that smaller droplets (2.5 pL) support rapid diffusion and homogeneous product distribution, whereas larger droplets (160 pL) exhibit interfacial product accumulation due to diffusion limitations. Complementary findings by Wei et al.^[^ ^2^ ^]^ further underscored that reduced droplet size, coupled with enhanced molecular diffusion, contributes directly to in‐drop reaction acceleration and spatial uniformity in product formation. Another key advantage of microdroplets lies in their exceptional tunability and manipulability. Parameters such as droplet size, composition, and interfacial properties can be precisely controlled to create programmable microenvironments that are tailored for specific physical and chemical transformations. For instance, the partitioning behavior of solutes and reagents across droplet interfaces can be selectively modulated for spatial segregation or enrichment.

While the attributes discussed above are broadly characteristic of microdroplets, the specific modes of reactivity can vary considerably depending on system design. Three general categories of microdroplet‐centered reactions have been proposed: in‐drop, on‐drop, and cross‐drop modes. These distinct regimes are especially relevant to the applications to be discussed in Sections 3 and 4, where microdroplets act as confined microreactors for chemical sensing and the synthesis of functional materials for water monitoring and remediation. In the in‐drop mode, all reactants are encapsulated within the droplet, providing a well‐defined and homogeneous microreactor. This configuration minimizes diffusion distances and enables rapid reaction kinetics, which makes it ideal for ultra‐fast material synthesis.^[^ ^69^ ^]^ The enclosed microenvironment also shields reactions from external perturbations, allowing for precise analytical detection of transient intermediates.^[^ ^70^ ^]^

When some reactants are confined inside droplets while others remain in the surrounding medium, two additional reactivity modes emerge. In the on‐drop (interfacial) mode, reactions are confined to the droplet interface, with minimal penetration of external reactants into the droplet core. This can be due to barriers such as solid shells,^[^ ^31^ ^]^ or low partition coefficients across the interface.^[^ ^71^ ^]^ The interface itself serves as a reactive boundary, often templating controlled growth and assembly of target materials.^[^ ^72^, ^73^ ^]^ In contrast, the cross‐drop mode involves the diffusion of external reactants or catalysts through the droplet interface, enabling reactions that depend on interfacial permeability. In such systems, reaction kinetics are governed by both partitioning behavior and diffusion rates across the droplet boundary.^[^ ^74^ ^]^ This mode combines the kinetic benefits of confinement with the flexibility of exchange, supporting the design of multistep catalytic cascades and continuous chemical processes.^[^ ^25^, ^26^ ^]^

Beyond reactivity classification, specific physical properties of microdroplets play pivotal roles in niche applications. For instance, the intrinsic plano‐convex geometry of microdroplets, combined with refractive index contrasts at liquid‐air or liquid‐liquid interfaces, confers strong optical focusing effects that significantly enhance light‐induced reactions.^[^ ^38^, ^75^ ^]^ Another example is the spontaneous generation of reactive oxygen species (ROS) at the air‐water interfaces of aqueous microdroplets. Multiple studies have reported the in situ formation of hydrogen peroxide (H_2_O_2_) and hydroxyl radicals (OH^•^).^[^ ^76^, ^77^, ^78^, ^79^, ^80^ ^]^ Recent investigations by Zhou et al.^[^ ^81^ ^]^ and Asserghine with co‐authors^[^ ^82^ ^]^ point to dissolved O_2_ as the primary source of these ROS. In contrast, Colussi and colleagues^[^ ^17^ ^]^ suggest that the partial dehydration of the interfacial OH^−^ and H^+^ ions plays a critical role in the spontaneous formation of H_2_O_2_. While the mechanistic origins of this phenomenon remain under active investigation, an expanding body of evidence highlights the key role of interfacial ROS in driving reaction acceleration. This distinct feature is leveraged in redox‐driven processes such as nitrogen fixation, which is further explored in Section 5.3.

Closely related to ROS generation is the role of interfacial electric fields (IEF), which are believed to modulate reaction pathways at microdroplet surfaces. Recent work by Krushinski and co‐workers^[^ ^83^ ^]^ reported that the IEF at the air‐water interface are significantly stronger than those at oil‐water interfaces. This higher IEF have been proposed to play a crucial role in accelerating chemical reactions,^[^ ^70^, ^84^ ^]^ potentially by organizing reactant molecules in configurations that lower the activation energy.^[^ ^67^, ^70^ ^]^ However, this hypothesis remains contested. Gong et al.^[^ ^85^ ^]^, in a combined experimental and computational study of the Diels‐Alder reaction between cyclopentadiene and acrylonitrile in water microdroplets, argued that the observed rate enhancement was instead driven by confinement effects and increased local reactant concentrations due to evaporation, with minimal contributions from IEF. These contrasting perspectives underscore the multifactorial and context‐dependent nature of microdroplet reactivity, reinforcing the need for continued mechanistic investigations to unravel the interplay between interfacial structure, field effects, and confinement dynamics. For readers interested in delving deeper into these complex mechanisms, several comprehensive review articles are available.^[^ ^2^, ^4^, ^70^, ^86^ ^]^

## 3 Functional Microdroplets as Platforms for Sensing and Chemical Analysis

Microdroplets have been increasingly integrated into chemical sensing and analysis, yielding versatile systems with high sensitivity, real‐time responsiveness, and tunable signal amplification.^[^ ^87^, ^88^ ^]^ A key distinction in their application lies in the dual roles they can adopt: as non‐reactive analyte concentrators or as reactive microreactors. The advanced functions of reactive microdroplets are often built upon the intrinsic characteristics that make non‐reactive microdroplets effective. Non‐reactive microdroplets enable rapid partition‐driven nanoextraction, effectively concentrating trace analytes for ultralow detection limits. Their curvature and refractive index also provide optical focusing effects that enhance signal intensity in spectroscopic or colormetric detection methods.^[^ ^89^, ^90^ ^]^ Reactive microdroplets leverage these foundational benefits while introducing additional capabilities, such as serving as confined chemical reactors or catalytic units to generate sensing in situ signals. These reactive systems can further transduce complex biochemical responses into easily detectable fluorescence or colorimetric readouts for localized and simplified quantification.^[^ ^91^, ^92^ ^]^

The growing utility of both non‐reactive and reactive microdroplets in sensing and chemical analysis is supported by their seamless integration with a wide range of analytical instruments, including mass spectrometry,^[^ ^93^, ^94^ ^]^ Raman spectroscopy,^[^ ^95^ ^]^ fluorescence microscopy,^[^ ^96^, ^97^ ^]^ and electrical sensors.^[^ ^80^, ^98^ ^]^ Their small and tunable volumes, compatibility with both static and flow‐based systems, and ability to function as isolated extractants, optical elements, or reaction compartments confer exceptional versatility. These features make microdroplets particularly ideal for detecting trace‐level analytes,^[^ ^42^, ^89^ ^]^ capturing short‐lived intermediates,^[^ ^4^ ^]^ and enabling lab‐on‐a‐chip diagnostics.^[^ ^99^ ^]^

### 3.1 Non‐Reactive Surface Microdroplets for Sensitive Detection

#### 3.1.1 Liquid and Complex Microdroplets for Extraction of Molecules, Biopolymers, and Microparticles

**Figure 2:**
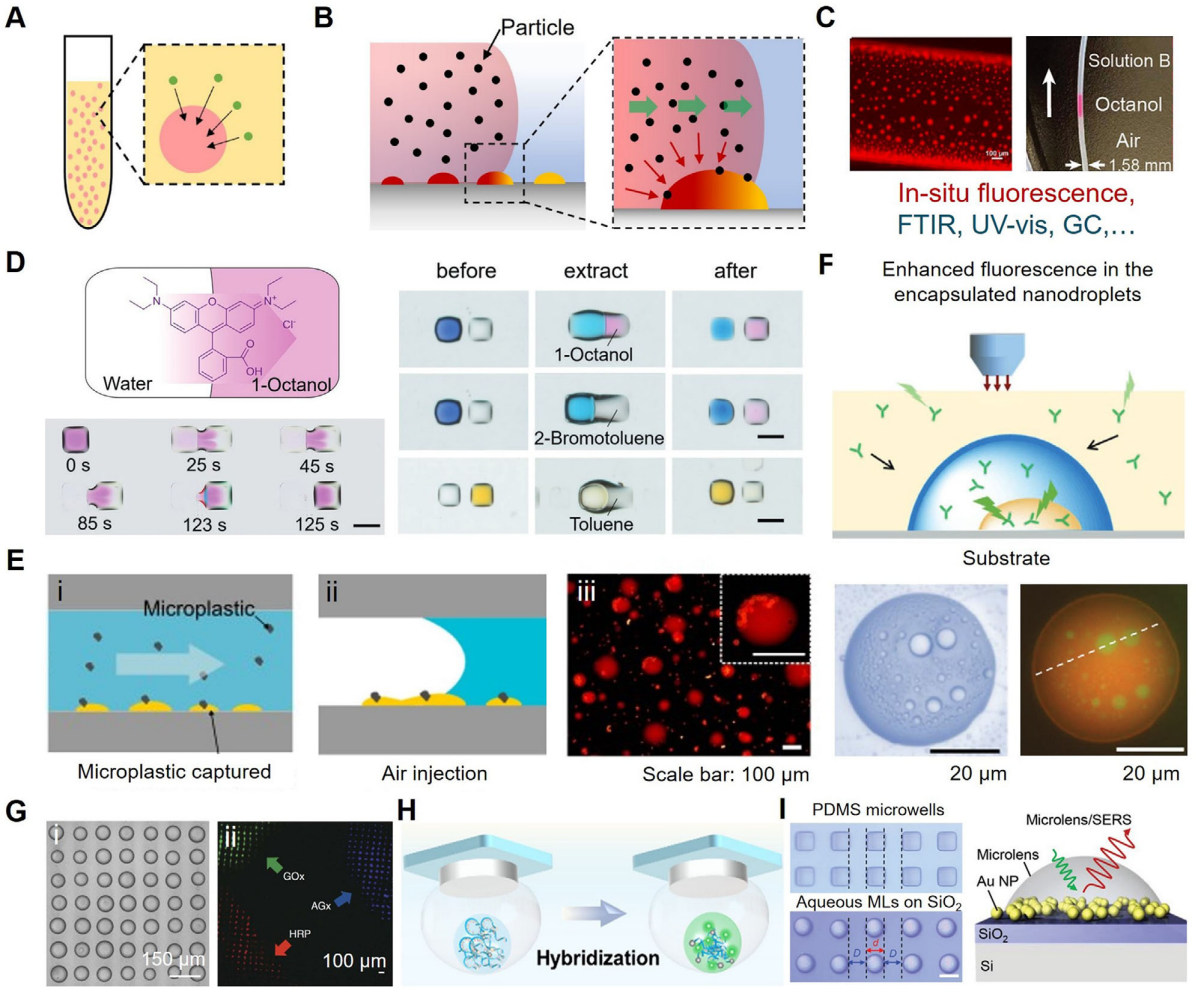
A) Schematic of the classical dispersive liquid–liquid microextraction (DLLME) process.^[^ ^42^ ^]^ Copyright 2022, American Institute of Physics (AIP). B) Schematic illustration showing the extraction of analytes from the sample flow into the surface nanodroplets. Reproduced with permission.^[^ ^104^ ^]^ Copyright 2021, Royal Society of Chemistry. C) A fluorescent image showing the octanol microdroplets pinned on the inner wall of the capillary tube.^[^ ^105^ ^]^ Copyright 2023, Elsevier, and the collected octanol in the capillary tube.^[^ ^101^ ^]^ Copyright 2022, American Chemical Society. D) A scheme and corresponding photos for the description of the extraction procedure of Rhodamine B from water (left) to 1‐octanol (right). Along with the extraction of varied analytes between neighboring droplets. (scale bar: 1 mm).^[^ ^106^ ^]^ Copyright 2023, Wiley. E) Schematic showcasing the capture of microplastics by surface nanodroplets in the (i) aqueous and (ii) air environment; (iii) A fluorescence microscopic image displaying microplastics on the nanodroplet interface. Reproduced with permission.^[^ ^35^ ^]^ Copyright 2024, American Chemical Society. F) Schematic demonstration of enhanced fluorescence intensity of molecules extracted into encapsulated nanodroplets from external solution, with bright‐field and fluorescence optical image of compartmentalized droplets in contact with a mixture of hydrophobic (red) and hydrophilic (green). Reproduced with permission.^[^ ^107^ ^]^ Copyright 2020, Wiley. G) (i) Optical microscopy images of acoustically patterned PDDA/ATP coacervate microdroplets; (ii) Fluorescence microscopy images of coacervate microdroplets containing varied analytes, including glucose oxidase (GOx), amyloglucosidase (AGx), or horseradish peroxidase (HRP).^[^ ^108^ ^]^ Copyright 2016, Nature. H) Schematic of the fluorescence generation by the accumulation of probe plus miRNA in the glucan phase and undergoing hybridization.^[^ ^109^ ^]^ Copyright 2024, Royal Society of Chemistry. I) Optical images of polydimethylsiloxane (PDMS) microwells (top) and transferred LiCl (3 m) aqueous solution microlenses (bottom), scale bar: 10 µm, and a schematic of the microlens‐substrate set‐up for detection.^[^ ^90^ ^]^ Copyright 2021, Wiley.

Nanoextraction, a process for concentrating and isolating analytes using droplets ranging from micro‐ to femtoliter volume,^[^ ^42^ ^]^ relies on partition differences between the droplet phase and the surrounding medium in the absence of chemical reactions. Owing to their high surface‐to‐volume ratio, short diffusion paths, and interfacial accumulation effects, microdroplets offer superior partitioning efficiency.^[^ ^100^, ^101^ ^]^ In contrast to conventional dispersive liquid‐liquid microextraction (DLLME), which typically relies on relatively large volumes of immiscible solvents and slow phase equilibration in bulk ternary mixtures,^[^ ^102^, ^103^ ^]^ nanoextraction uses significantly smaller extractant volumes. This not only improves extraction efficiency, but also mitigates the use of hazardous solvents, offering a more environmentally sustainable alternative.^[^ ^42^, ^104^ ^]^ There are three principal approaches to droplet‐based nanoextraction: single‐drop extraction, dispersive liquid‐liquid extraction in microfluidic systems (**Figure** **2**A), and surface‐confined micro‐/nanodroplet extraction (Figure 2B).^[^ ^42^ ^]^ Among these, surface micro‐/nanodroplets immobilized on flat or curved substrates offer distinct advantages, including enhanced stability under flow conditions and compatibility with the in situ optical observation. These features make them particularly well‐suited for rapid spectroscopic sensing and colorimetric assays. The subsequent discussion of this section will primarily focus on non‐reactive nanoextraction techniques employing surface‐confined microdroplets.

To integrate nanoextraction with fluorescence detection, Wu et al.^[^ ^105^ ^]^ developed a capillary‐based surface nanodroplet system in which extractant droplets (e.g., octanol) were grown along the inner wall of a microchannel (Figure 2C). As the sample solution flowed through the capillary, the target analytes were selectively partitioned into oil nanodroplets due to their higher favorable partition coefficients. This system achieved a 3‐order‐of‐magnitude enhancement in the fluorescence signal and a 20‐fold reduction in detection limits compared to conventional DLLME. The method proved to be especially suitable for continuous or periodically programmed workflows, offering high enrichment efficiency and scalability. Wiedmann et al.^[^ ^106^ ^]^ combined droplet‐based nanoextraction with optical detection and matrix‐assisted laser desorption/ionization mass spectrometry (MALDI‐MS). Their static, parallel nano‐liquid‐liquid extraction system utilized pre‐patterned arrays of aqueous and organic nanoliter droplets (Figure 2D). Analyte extraction occurred at the droplet‐droplet interface, triggered by controlled solvent evaporation. This strategy resulted in a reproducible extraction efficiency of ≈60% as a robust high‐throughput purification method.

Beyond single‐interface extraction between microdroplets and the surrounding flow, recent innovations in droplet architecture have broadened the functional capabilities of nanoextraction. Li and colleagues^[^ ^107^ ^]^ developed a composite structure comprising nanodroplets encapsulated within a larger microdroplet, enabling dual‐phase extraction of both hydrophilic and hydrophobic analytes. The refractive index contrast between the internal and external phases created curved interfaces that acted as optical lenses, locally amplifying fluorescence signals by up to 60 times (Figure 2F). This strategy not only facilitated efficient analyte partitioning but also provided built‐in optical enhancement, which offers a multifunctional platform for simultaneous extraction and signal amplification. Together, these studies underscore the versatility of micro‐ and nanodroplet systems in chemical and environmental sensing. Whether implemented as flow‐through systems or static arrays, these architectures support a wide range of analytical functions, including enrichment of emerging contaminants, enhancement of detection sensitivity, and development of hybrid sensing modalities.

Nanoextraction techniques have also been adapted for the enrichment of colloids and particles hard to capture from the aqueous phase, such as microplastics, from the flowing aqueous phase, enabling subsequent characterization and size analysis. For instance, Faramarzi et al.^[^ ^35^ ^]^ demonstrated this approach using octanol surface nanodroplets generated within a microfluidic device (Figure 2E‐i) to selectively capture microplastics from flowing water, achieving a capture efficiency of 82.1%. The effectiveness and long‐term stability of this system are attributed to the significantly higher adhesion energy between the microplastic particles and the droplet‐water interface, compared to the energy required for detachment. This strong interfacial binding allowed the retention of microplastic particles even after the aqueous phase was displaced by air (Figure 2E‐ii), allowing for subsequent in situ analysis. Detailed characterization of microplastic size, shape, and chemical composition was then performed using fluorescence microscopy (Figure 2E‐iii) and Raman spectroscopy.

Coacervate microdroplets, formed through liquid‐liquid phase separation (LLPS) of oppositely charged polyelectrolytes or biomolecules in aqueous media, represent a distinct class of aqueous phase extraction systems that complement conventional nanoextraction techniques.^[^ ^110^, ^111^ ^]^ Unlike conventional liquid nanodroplets based on immiscible organic solvents, coacervates offer a dense, charge‐rich interior that enables the selective partitioning of biomolecules and ionic species.^[^ ^112^ ^]^ Their entirely aqueous composition provides excellent compatibility with biological samples, avoiding the cytotoxic effects commonly associated with organic extractants.^[^ ^113^ ^]^ These advantages have led to their increasing use in biomedical sensing, diagnostics, and analytical sample preparation.^[^ ^114^, ^115^, ^116^, ^117^ ^]^ For instance, Tian and collaborators^[^ ^108^ ^]^ engineered periodic 2D arrays of polydiallydimethylammonium chloride/adenosine 5'‐triphosphate (PDDA/ATP) coacervate microdroplets (Figure 2G‐i). These coacervate arrays selectively captured a range of analytes, including dyes, proteins, enzymes, and nanoparticles (Figure 2G‐ii), facilitating the collection and detection of spatially patterned analytes. More recently, the utility of coacervate‐based nanoextraction has expanded into biomedical applications. Zha et al.^[^ ^109^ ^]^ developed an integrated minipillar array featuring an aqueous two‐phase system for the enrichment and fluorescence‐based detection of ultra‐trace microRNAs (miRNAs). Target miRNAs labeled with fluorescent probes preferentially partitioned into the dextran‐rich phase (Figure 2H), resulting in more than 200‐fold signal amplification.

#### 3.1.2 Microdroplets as Optical Signal Amplifier for Chemical Analysis

Surface‐bound microdroplets can inherently function as optical elements, leveraging their plano‐convex geometry and the refractive index contrast at the liquid–air or liquid–liquid interface to focus incident light. Acting as converging microlenses, these microdroplets concentrate incident light into tightly confined focal volumes, thereby amplifying optical signals from analytes within or surrounding the droplet.^[^ ^75^ ^]^ This optical enhancement leads to improved detection sensitivity in spectroscopic applications. Importantly, the focal length of microdroplet lenses is tunable by modifying the contact angle, which can be precisely controlled through parameters such as droplet volume, liquid composition, surface wettability, and substrate morphology.^[^ ^90^ ^]^ Fabrication of such microdroplets is straightforward and cost‐effective, often relying on simple droplet deposition or controlled nucleation on modified substrates. These features position microdroplets as adaptable and scalable components for advanced optical sensing technologies.

Surface‐enhanced Raman spectroscopy (SERS), renowned for its high sensitivity and molecular specificity, relies on plasmonic nanoparticles to amplify Raman scattering signals from target molecules.^[^ ^118^ ^]^ These plasmonic nanostructures (e.g., Ag or Au) significantly enhance Raman signals through a combination of electromagnetic and chemical mechanisms, enabling the detection of analytes at extremely low concentrations.^[^ ^42^, ^100^, ^119^ ^]^ Tsao et al.^[^ ^89^ ^]^ utilized water microdroplets deposited on customized hydrophobic Ag nanoparticle‐decorated substrates to form plano‐convex lenses that improved both laser focusing and scattered light collection. Experimentally, this led to a 2.7‐fold increase in Raman intensity compared to measurements taken without microdroplets. Similarly, Kim and colleagues^[^ ^90^ ^]^ introduced LiCl aqueous microdroplet arrays fabricated via polydimethylsiloxane (PDMS) microwells for gas‐phase SERS detection (Figure 2 (I)). These microdroplets acted simultaneously as gaseous analyte collectors and optical microlenses to amplify Raman scattering, which led to rapid detection of dimethyl methylphosphonate vapor at concentrations as low as 0.8 ppm within 5 s. Simulations results confirmed that the microdroplet‐SERS system achieved an electric field enhancement of up to 28.1 times.

### 3.2 Reactive Surface Microdroplets for Analytical Detection

In contrast to their non‐reactive counterparts, reactive nano‐/microdroplets not only concentrate species but also facilitate accelerated chemical reactions within their confined microenvironments. This dual functionality allows real‐time analysis of transient intermediates,^[^ ^120^, ^121^, ^122^ ^]^ continuous monitoring of dynamic reaction systems,^[^ ^94^, ^123^ ^]^ and selective signal amplification of detection signals to increase analytical sensitivity.^[^ ^124^ ^]^ These advantages stem from the unique physicochemical characteristics discussed in Section 2. The following section highlights recent developments in the deployment of reactive nano‐/microdroplets for advanced analytical applications, and illustrates how confinement‐enhanced chemistry can be harnessed for sensitive and selective detection.

**Figure 3:**
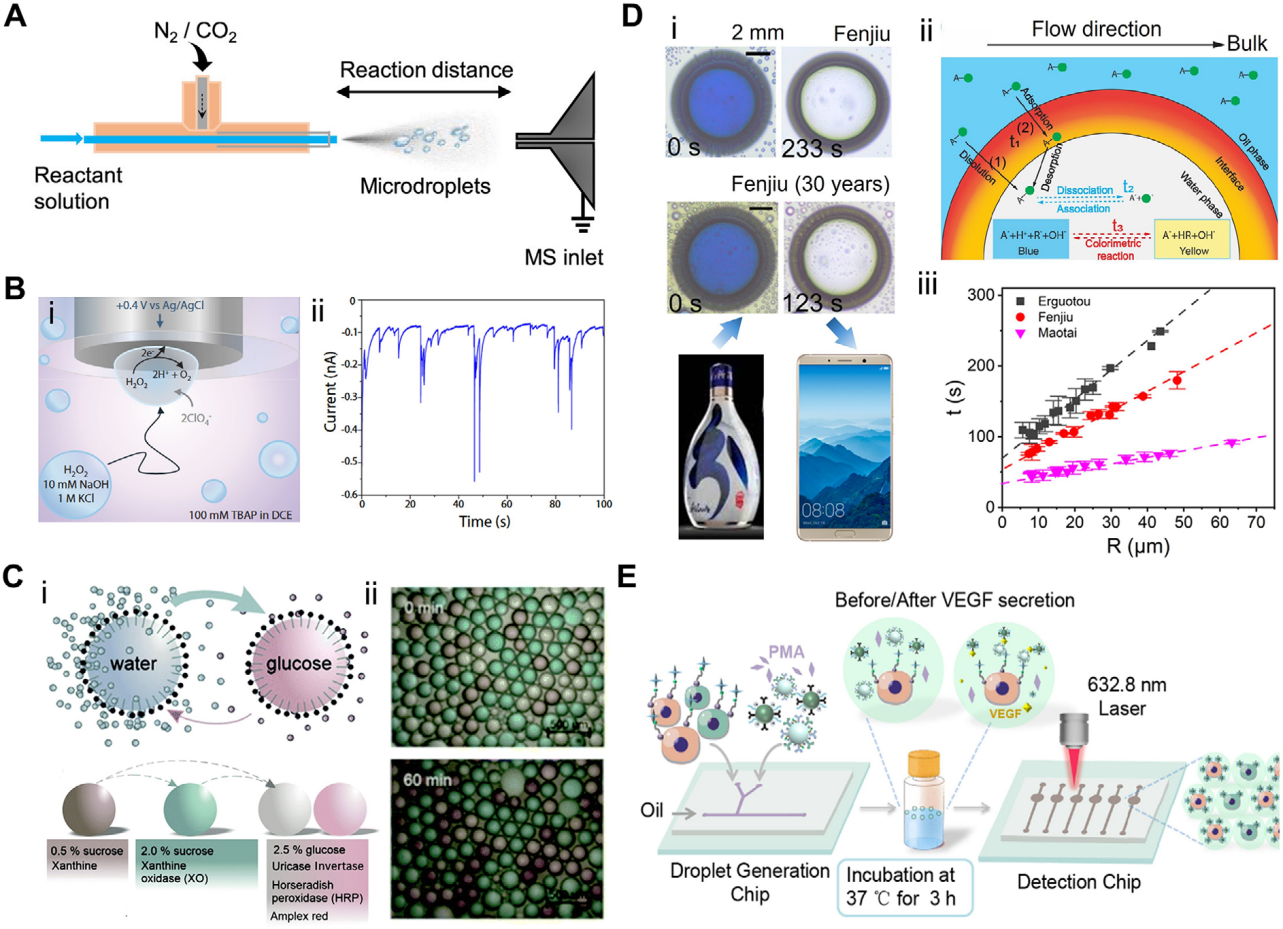
A) Schematic of the experimental setup of sprayed droplets coupled mass spectrum detection.^[^ ^126^ ^]^ Copyright 2024, American Chemical Society. B) (i) Schematic showing the collisions of emulsion droplets on Pt microelectrode and subsequent oxidation of H_2_O_2_; (ii) The characteristic transient of currents resulted from the collision of a single droplet with the microelectrode (rapid rise followed by an exponential decay).^[^ ^80^ ^]^ Copyright 2024, National Academy of Sciences. C) (i) Schematics of the color changes during enzymatic cascades in aqueous microdroplet; (ii) Time‐dependent evolution of the cascade reaction progress tracked via optical images of aqueous microdroplets. Reproduced with permission.^[^ ^128^ ^]^ Copyright 2021, Royal Society of Chemistry. D) (i) Decolorizatoin process in single surface nanodroplets for two Chinese spirits with varied hoard years, which can be monitored by the portable device; (ii) Sketch of nanoextraction and colorimetric reaction in surface microdroplets; (iii) The decoloration time (t) as a function of the nanodroplet lateral radius (R). Reproduced with permission.^[^ ^124^ ^]^ Copyright 2020, American Chemical Society, and^[^ ^129^ ^]^. Copyright 2022, American Chemical Society. E) Workflow sketch of Surface‐enhanced Raman Spectroscopy detection of single‐cell‐secreted growth factors in microdroplets based on the cell surface bioconjugation. Reproduced with permission.^[^ ^91^ ^]^ Copyright 2022, American Chemical Society.

Microdroplet‐based reaction platforms have been integrated with high‐precision analytical techniques to probe ultrafast reaction dynamics.^[^ ^94^, ^120^, ^121^, ^122^, ^125^ ^]^ For instance, Chen's group^[^ ^126^ ^]^ developed a custom mass spectrometry (MS)‐coupled spraying setup (**Figure** **3**A) to detect transient –CH_2_CN species formed spontaneously from acetonitrile at the air‐water interface of microdroplets. By precisely directing the spray into the MS inlet, short‐lived intermediates ‐ typically undetectable under bulk conditions ‐ could be captured and characterized in real time. Similarly, Huang et al.^[^ ^127^ ^]^ utilized desorption electrospray ionization‐MS to monitor the accelerated functionalization of bioactive molecules within microdroplets generated by high‐voltage spray. This setup enabled the collection of both product‐specific MS data and spatial intensity maps, offering high‐throughput insights into reaction efficiency with the nano‐gram sample input under varying conditions. In both cases, microdroplets served as self‐contained microreactors, while MS provided sensitive, structure‐specific detection of intermediates and products, underscoring the synergy between microdroplet chemistry and advanced analytical tools.

The utility of reactive microdroplets extends beyond chemical analysis and includes a broad range of analytical modalities. For example, Krushinski and Dick^[^ ^80^ ^]^ employed stochastic electrochemistry to investigate the spontaneous formation of H_2_O_2_ in aqueous microdroplets suspended in oil (Figure 3B‐i). As individual microdroplets contacted a microelectrode, each produced a discrete current spike (Figure 3B‐ii), enabling label‐free and single‐droplet level detection of reactive species in real time. In another study, Pavlovic et al.^[^ ^128^ ^]^ designed a reactive microdroplet network to monitor multi‐step enzymatic cascade reactions. Aqueous microdroplets containing specific enzymes or substrates were generated by microfluidic emulsification in tetradecane oil. The reaction progress was tracked using droplet size changes and optical microscopy, which revealed colorimetric shifts associated with osmotic activity (Figure 3C‐i). The completion of the cascade was confirmed by the appearance of pink/red fluorescence from resorufin, visualized by fluorescence microscopy (Figure 3C‐ii). This example illustrates the potential of reactive microdroplet networks for programmable biochemical sensing and signal amplification.

To achieve dynamic and rapid detection of organic acids, Wei et al.^[^ ^124^ ^]^ designed a surface nanodroplet‐based technique that integrates nanoextraction with colorimetric sensing using halochromic indicators (Figure 3D‐i). These aqueous surface nanodroplets selectively extracted organic acids from the surrounding oil phase. The enriched analytes triggered a visible decoloration response, governed by the solubility and dissociation properties of the target acids (Figure 3D‐ii).^[^ ^129^, ^130^ ^]^ Such an integrated sensing system enabled the identification of over 27 distinct organic acids while minimizing reagent consumption.^[^ ^124^ ^]^ Notably, the decoloration time (*t*) displayed a linear dependence on droplet size and showed brand‐specific variation when applied to Chinese spirits of different brands and hoard time (Figure 3D‐iii). This technique has potential applications in beverage anti‐counterfeiting or flavor profiling and can even be deployed using a smartphone camera. (The concept of anti‐counterfeiting droplets was inspired by Professor Lei Jiang, Chinese Academy of Sciences.)

Several integrated systems leveraged the advantages of microfluidics for continuous microdroplet generation to enhance signal detection. For instance, Cong and co‐workers^[^ ^91^ ^]^ developed a microfluidic platform that couples microdroplet arrays with SERS for single‐cell cytokine profiling (Figure 3E). In this system, immuno‐sandwich probes anchored to cell surfaces induced aggregation of plasmonic nanoparticles, generating SERS hot spots that enabled sensitive and specific detection of secreted vascular endothelial growth factor. Continuous droplet generation and automated spectral acquisition facilitated high‐precision digital analysis. In a related advancement, Ho et al.^[^ ^92^ ^]^ presented a droplet‐based microfluidic SERS system for the label‐free detection of exosomes. In their design, aptamer‐bound Au nanoparticles selectively aggregated in response to target exosomes, forming transient SERS‐active sites. The system achieved a detection limit of 4.5 log_10_ particles per mL in plasma within just 5 min. These compact and integrated platforms highlight the potential of droplet‐based SERS technologies for medically and clinically relevant diagnostics.

## 4 Reactive Microdroplet‐Driven Synthesis of Multifunctional Materials for Aquatic Pollutant Monitoring and Decontamination

Reactive microdroplets serve as novel microreactors for the tailored synthesis of functional materials, offering a powerful approach to address key challenges in the quantification and removal of contaminants from aqueous environments.^[^ ^131^, ^132^ ^]^ The controlled formation and manipulation of microdroplets allow the creation of well‐defined microenvironments that facilitate precise tuning of material properties.^[^ ^133^, ^134^, ^135^ ^]^ This section highlights recent progress in microdroplet‐assisted synthesis of functional materials, including plasmonic nanoparticles for SERS detection, polymeric microparticles, metal oxides, metals, and metal–organic frameworks, with demonstrated applications in water treatment and wastewater remediation.

### 4.1 Aquatic Pollutant Monitoring with Microdroplet‐Derived Advanced Materials

#### 4.1.1 Plasmonic Nanostructures from Microdroplets for Surface‐Enhanced Raman Spectroscopy (SERS)

Ultrasensitive detection of trace analytes is essential across environmental monitoring, biomedical diagnostics, and forensic science.^[^ ^136^ ^]^ Among available analytical methods, SERS has emerged as a leading technique, offering rapid and non‐destructive detection with single‐molecule sensitivity.^[^ ^137^, ^138^ ^]^ The performance of SERS depends critically on the morphology of plasmonic nanoparticles, their interparticle spacing (which governs hot spots distribution), surface homogeneity, and the efficiency of analyte‐nanoparticle interactions.^[^ ^34^, ^139^, ^140^ ^]^ One of the challenges in plasmonic nanoparticles preparation is aggregation and instability of nanoparticles during storage and utilization.^[^ ^141^, ^142^ ^]^ Microdroplets offer powerful strategies for the synthesis of plasmonic nanostructures customized for SERS applications due to their spatial confinement and ease of precise assembling.^[^ ^25^, ^143^ ^]^

Microdroplet‐templated synthesis offers a fast, reproducible, and scalable alternative to conventional lithographic or wet‐chemical fabrication methods. Operating under ambient conditions, this approach allows precise control over parameters such as droplet size, precursor concentration, and interfacial templating. These tunable features allow for the tailoring synthesis of plasmonic nanostructures without the need for complex processing or hazardous reagents. For example, Lee et al.^[^ ^144^ ^]^ demonstrated a method in which aerosolized droplets containing Au precursors spontaneously nucleated and grew into plasmonic nanostructures at the air‐liquid interface. Remarkably, this synthesis proceeded without any reducing agents or external templates, relying solely on interfacial effects to define nucleation and growth. The resulting 3D dendritic nanostructures −≈7 µm wide and over 2 µm long ‐ exhibited features highly favorable for SERS enhancement, highlighting the potential of droplet‐mediated synthesis in producing high‐performance sensing substrates.

**Figure 4:**
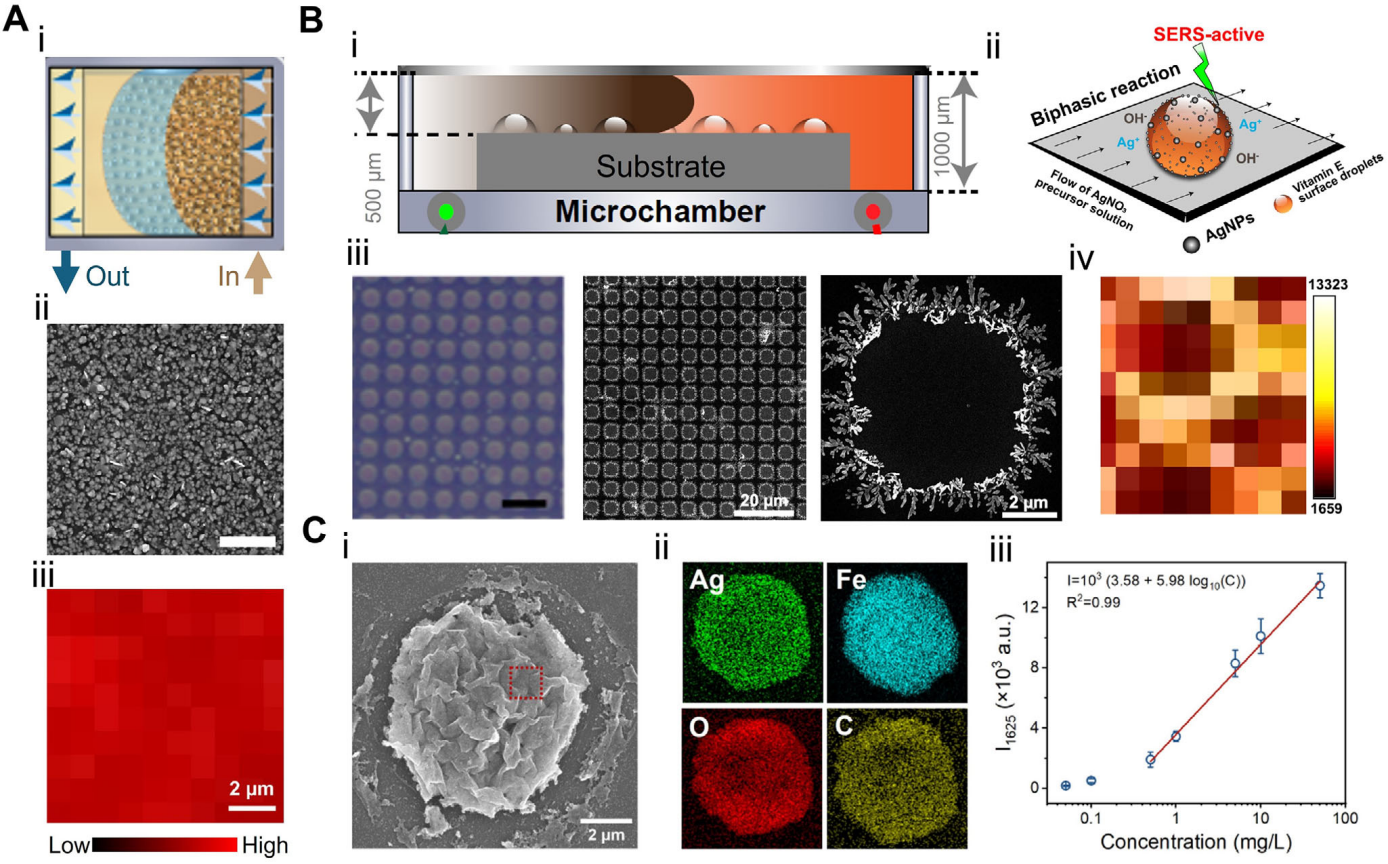
A) (i) Schematic illustrating the formation of a VE film and surface nanodroplets on PDMS via a solvent exchange process in a 3D‐printed microchamber; (ii) FESEM image of Ag nanoparticles formed on the PDMS substrate; (iii) 2D Raman mapping of Rhodamine 6G (R6G) at 612 cm^−1^ on the Ag nanoparticles–PDMS substrate. Reproduced with permission.^[^ ^145^ ^]^ Copyright 2024, American Chemical Society. B) (i) Schematic overview of the solvent exchange process; (ii) Biphasic reaction mechanism between VE nanodroplets and Ag^+^ ions for Ag nanostructure synthesis; (iii) VE surface nanodroplets on a patterned substrate and corresponding scanning electron microscopy (SEM) image of Ag nanoring arrays; (iv) 2D Raman intensity mapping of the Ag nanorings. Reproduced with permission.^[^ ^146^ ^]^ Copyright 2023, Elsevier. C) (i) SEM image of a single MIL‐100/Ag dome; (ii) Elemental mapping of the MIL‐100/Ag structure; (iii) Linear correlation between methylene blue concentration and Raman intensity at 1625 cm^−1^. Reproduced with permission.^[^ ^31^ ^]^ Copyright 2025, Elsevier.

In another study, Yan et al.^[^ ^145^ ^]^ introduced a microfluidic surface nanodroplet platform to streamline the synthesis of Ag nanoparticles directly onto plain PDMS substrates (**Figure** **4**A‐i). The nanodroplet‐hosted reactions facilitated a diffusion‐dominated nucleation process, yielding dendritic Ag structures rich in SERS hot spots (Figure 4A‐ii). The low surface energy of PDMS allowed the coexistence of surface droplets and a liquid film of the reducing agent, promoting uniform deposition and signal homogeneity (Figure 4A‐iii). Raman mapping over 100 random points showed relative standard deviations (RSD) as low as 8.5%, indicating excellent signal reproducibility. This substrate achieved detection limits down to 10^−11^ m for Rhodamine 6G and 10^−7^ m for adenine, and successfully identified characteristic peaks for 12 different biological toxins. Compared to aerosol‐based techniques, surface nanodroplet approaches offer direct fabrication of SERS‐active substrates, simplifying integration of plasmonic materials with small‐volume analyte detection systems.^[^ ^147^ ^]^

To further improve SERS reproducibility, Kanike and colleagues^[^ ^146^ ^]^ engineered microring arrays of dendritic Ag nanoparticles by reacting vitamin E (VE) nanodroplets confined to circular hydrophobic patterns with flowing aqueous solution containing Ag^+^ ions (Figure 4B‐i,ii). Nucleated nanoparticles were preferentially deposited on the three‐phase contact line and formed highly uniform ring structures (Figure 4B‐iii). By tuning ring thickness and nanoparticle morphology, SERS hot spots were localized along the rim of each microring, resulting in consistent signal amplification across large areas (Figure 4B‐iv). This substrate achieved detection limits of 10^−12^ m for rhodamine 6G and 10^−4^ m for tetrahydrocannabinol (THC) in saliva, with stable SERS performance over surfaces larger than 60 cm^2^. The integration of the microring substrate into a 3D‐printed microchamber enabled real‐time and low‐volume analysis while preserving detection sensitivity and precision.

Building on this droplet approach, Wu et al.^[^ ^31^ ^]^ functionalized metal–organic frameworks (MOF) domes with Ag nanoparticles through sequential interfacial reactions. Scanning electron microscope imaging revealed ≈20 nm Ag nanoparticles uniformly anchored to the textured MOF surfaces (Figure 4C‐i,ii). The hierarchical porosity of the MOF facilitated efficient analyte adsorption and facilitated synergistic interactions between pollutants and the Ag nanoparticles‐decorated substrate. Raman mapping confirmed spatially uniform signal distribution across MOF domains, enabling reliable and real‐time quantification of pollutants during photocatalytic degradation (Figure 4C‐iii).

**Figure 5:**
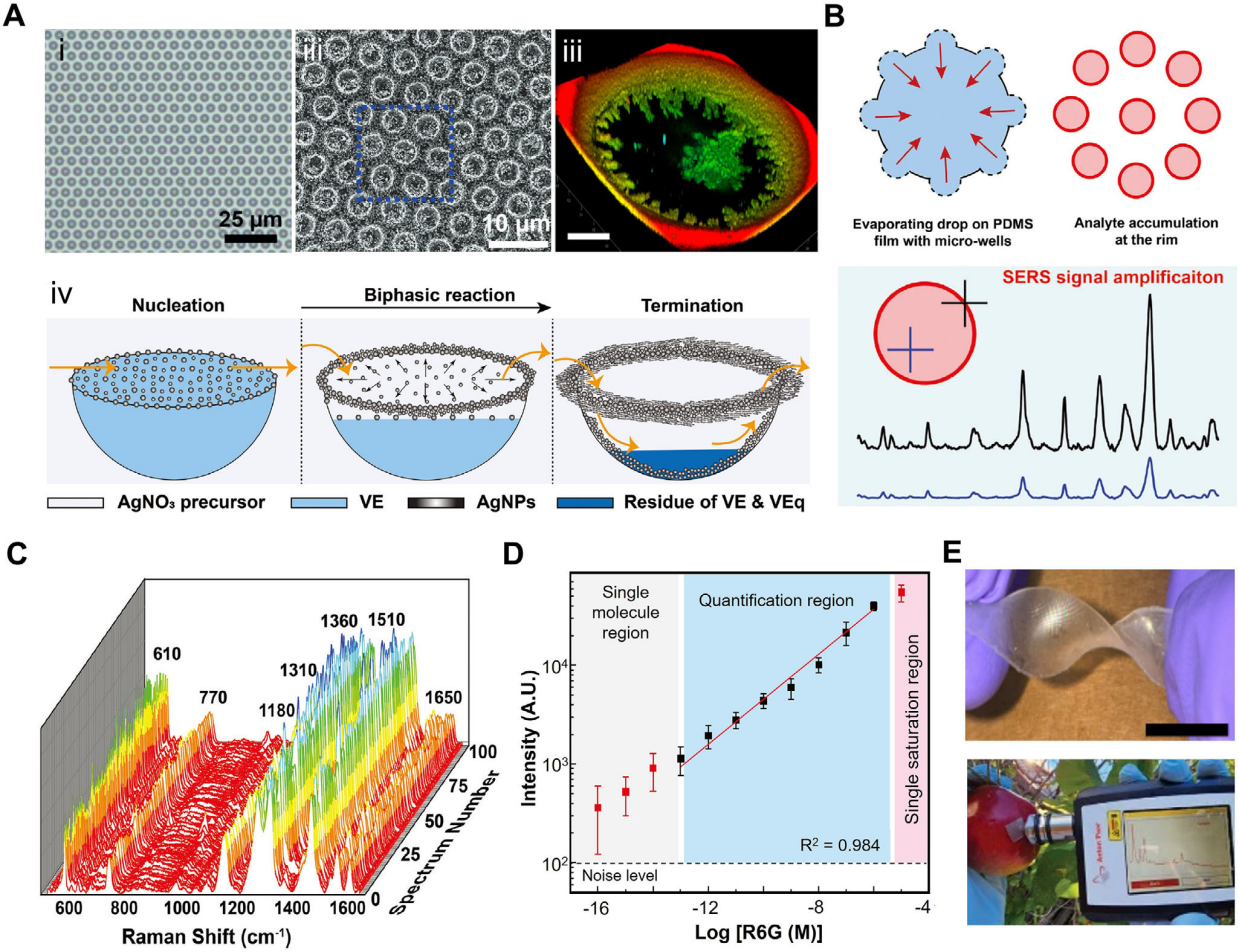
A) (i) Microwell array on the surface of the PDMS film; (ii) SEM image of Ag nanostructures formed within the microwells; (iii) Confocal image of VE droplets confined in microwells (scale bar: 200 µm); (iv) Schematic illustrating the nucleation and growth of Ag nanoparticles at the VE droplet interface in the presence of AgNO_3_ precursor. B) Illustration of droplet evaporation on the microwell‐patterned PDMS film, with arrows marking the receding contact line that leads to stronger SERS signals at the rim. C) SERS spectra acquired from 100 different locations across the plasmonic PDMS film. D) Linear relationship between SERS intensity at 612 cm^−1^ and Rhodamine 6G concentration (error bars represent >10 spectra from varied positions). E) Demonstration of the flexibility and mechanical robustness of the plasmonic PDMS film (scale bar: 2 cm), applied on an apple surface for in situ pesticide detection. Reproduced with permission.^[^ ^34^ ^]^ Copyright 2024, Wiley.

To fabricate transparent and flexible SERS films suitable for wearable sensing, Kanike et al.^[^ ^34^ ^]^ refined the surface nanodroplet strategy by confining Ag dendrites growth within PDMS microwell arrays (**Figure** **5**A–E). The geometry of microwells induced nanodroplet pinning, which ensured uniform spatial localization and prevented coalescence. Uniform VE nanodroplets formed within these microwells and served as microreactors, where laser confocal microscopy revealed the dynamic in situ formation of intricate Ag nanoparticle networks. These evolved into dense dendritic structures, particularly enriched along the microwell rim (Figure 5B), where abundant ultra‐dense hot spots amplified SERS signals. The mapping of 100 SERS spectra for Rhodamine 6G (10^−7^ m) across the film confirmed uniform sensitivity, and the signal scaled with analyte concentration across a wide range (10^−6^–10^−13^ m). The final SERS substrate combined both transparency and flexibility, which allowed in situ pesticide detection on curved surfaces such as fruit skins, significantly expanding their utility for field‐deployable and wearable sensing technologies.

#### 4.1.2 Artificial Compound Eyes from Microdroplets for Optical Signal Amplification

**Figure 6:**
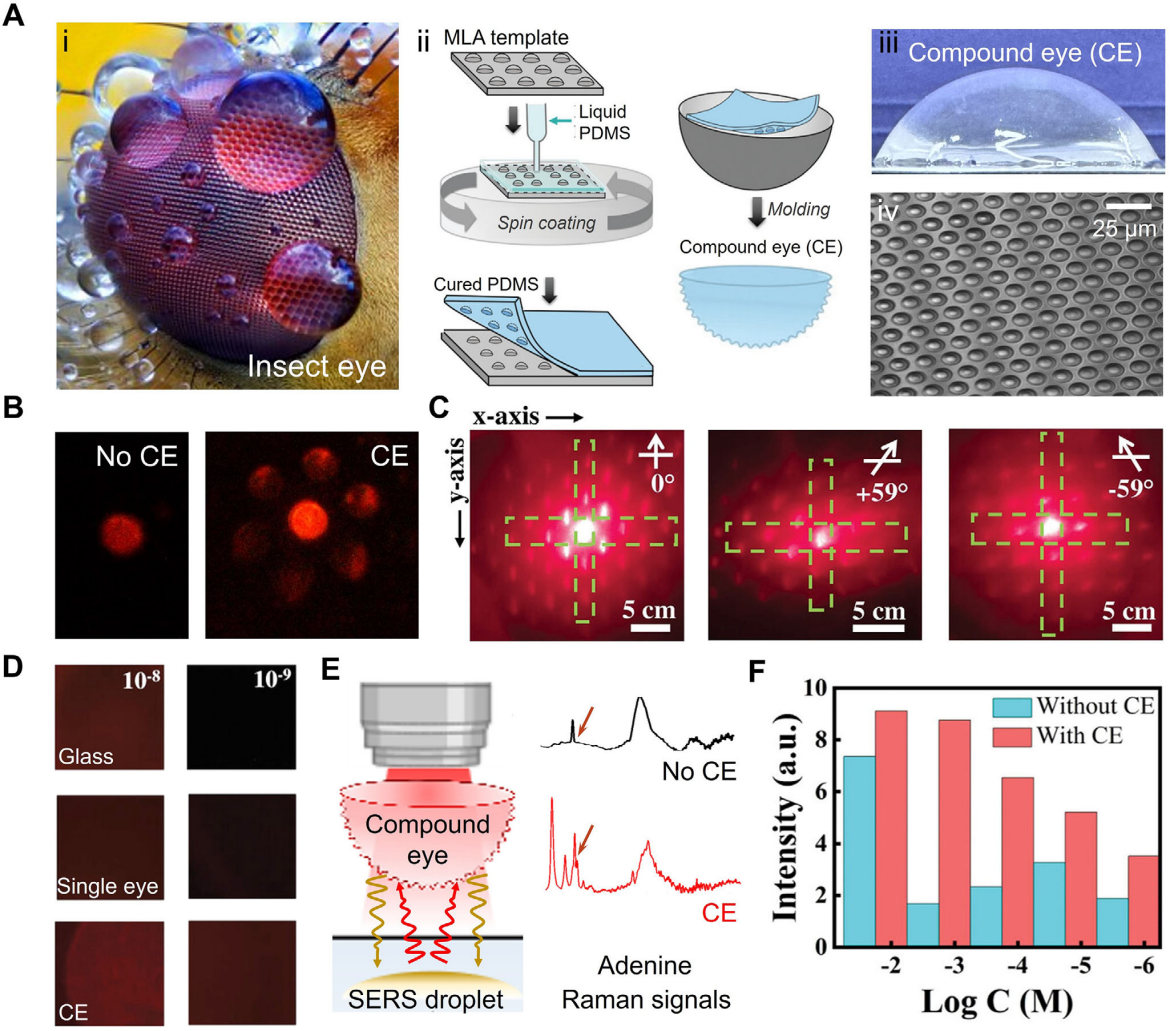
A) (i) Optical image of an insect eye; (ii) Schematic of compound eye (CE) fabrication using a microlens array (MLA) template derived from surface microdroplets; (iii) Photograph of the resulting CE featuring microwells on a curved surface; (iv) SEM image of the CE top surface. B) Optical projection of a glass bead with and without CE. C) Focal points observed at 0° (top), +59° (middle), and −59° (bottom) using a 5‐mm CE. D) Fluorescence signals of Rhodamine 6G (10^−8^ and 10^−9^ m) imaged through glass, single lens (no ommatidia), and CE. E) Application of CE for Surface‐Enhanced Raman Spectroscopy (SERS) detection and representative Raman spectrum of adenine. F) Comparison of SERS signal intensity for different adenine concentrations, with and without CE. Reproduced with permission.^[^ ^36^ ^]^ Copyright 2024, Wiley.

Microdroplet templating has also been applied in the fabrication of bio‐inspired compound eyes (CE), mimicking the visual architecture of insects like dragonflies, moths, and mosquitoes (**Figure** **6**A‐i),^[^ ^148^, ^149^ ^]^ for amplifying optical signals in aquatic pollutant detection. In contrast to conventional fabrication techniques such as laser writing,^[^ ^150^, ^151^, ^152^ ^]^ or mechanical machining,^[^ ^153^, ^154^, ^155^ ^]^ which require expensive equipment and energy‐intensive processing, microdroplets provide a low‐energy and high‐precision templating approach for the formation of ommatidia on compound eye surfaces. In a recent study, a CE was fabricated by combining a PDMS dome with concave microlens arrays produced through soft lithography. The concave microlens array itself was templated from in situ polymerization of microdroplets on a chemically patterned substrate (Figure 6A‐ii).^[^ ^36^ ^]^ The resulting CE exhibited a macrodome morphology, with a tunable diameter ranging from 5 to 90 mm depending on the mold used (Figure 6A‐iii). Each ommatidium measured ≈5 µm in diameter with 1.1 µm spacing (Figure 6 (A‐iv)), and the surface of the CE contained up to 1.9 million ommatidia, corresponding to the number of microdroplets formed during fabrication.

This CE enabled clear imaging of letters and fluorescent microsphere glass beads, with enhanced focusing capability compared to simple eyes (Figure 6B). Its architecture featured densely packed ommatidia arranged on a curved surface, providing a wide peripheral field‐of‐view (118°) (Figure 6C). The combination of strong focusing capability and a wide field‐of‐view makes compound eyes ideal optical elements for enhancing fluorescence or SERS signals in ultrasensitive detection of analytes in aqueous solution. This system achieved a detection limit for fluorescent dyes, such as Rhodamine 6G, in aqueous solution as low as 10^−12^ m, representing a 100‐fold and 10 000‐fold improvement over bare glass and single‐lens setups, respectively (Figure 6D). When placed between the objective and the analyte droplet, the CE amplified SERS signal intensity by 3 times (Figure 6E,F). Moreover, the spatial multiplexing enabled by the dense hot spots array holds potential for integration with machine learning and AI, offering new possibilities for high‐content sensing and intelligent signal decoding.

### 4.2 Water Decontamination Powered by Functional Materials from Microdroplets

#### 4.2.1 Mobile versus Immobilized Microdroplet Reactors

Microdroplet‐based synthesis offers precise control over morphology, size distribution, and surface functionality, which are critical parameters for optimizing the performance of polymeric materials in water treatment applications. One of the key advantages of this approach is its ability to produce particles with narrow and tunable size distributions, ensuring predictable behavior in adsorption, separation, and catalysis.^[^ ^18^, ^156^, ^157^ ^]^ Moreover, the mobility and structural flexibility of microdroplet‐fabricated systems facilitate surface modifications, broadening their utility as encapsulating agents, catalyst supports, or active additives in environmental remediation.^[^ ^158^ ^]^

**Figure 7:**
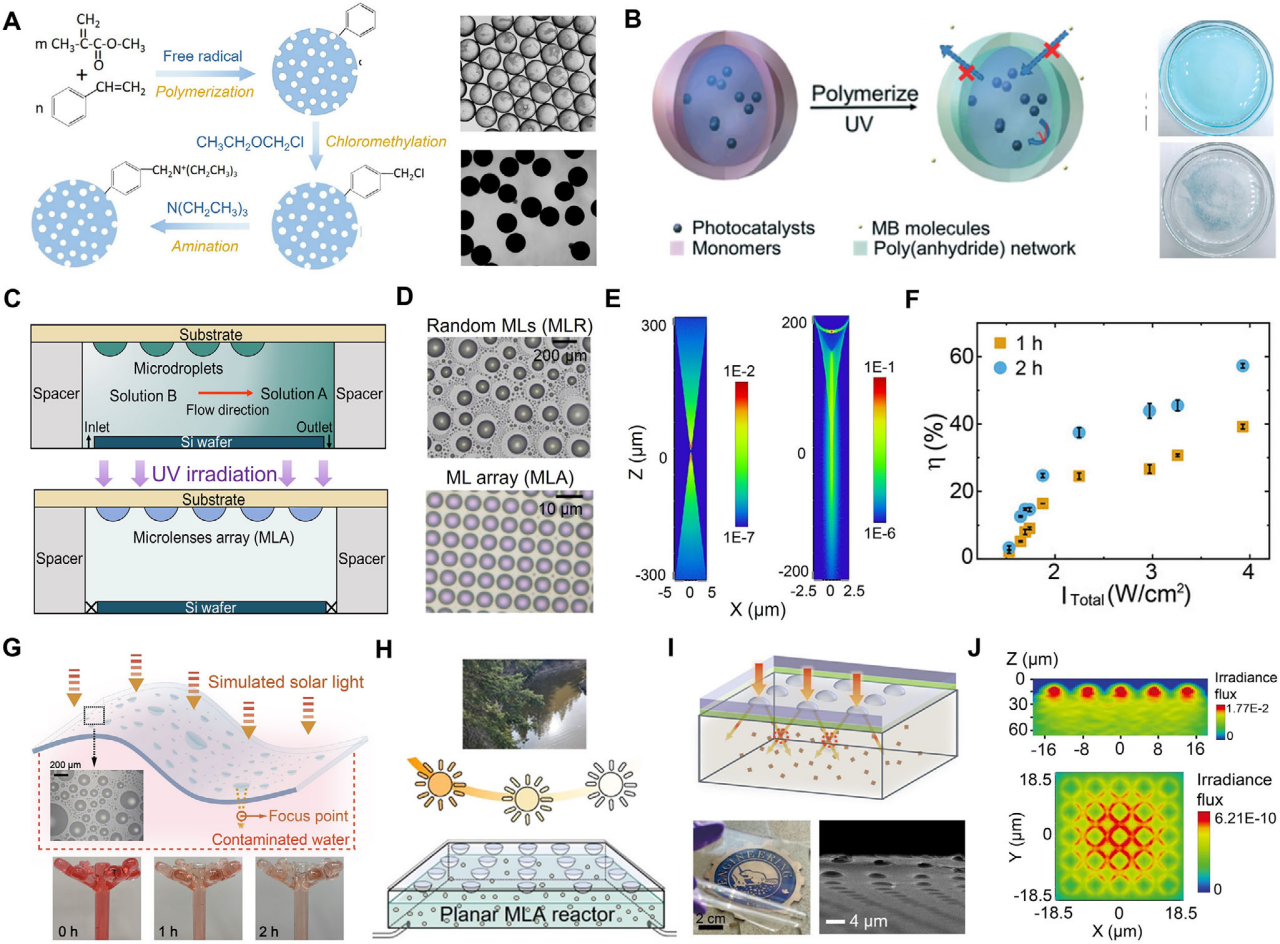
A) Schematic of the formation of triethylamine‐functionalized polystyrene/methyl methacrylate (PMMA) microspheres from microdroplets, with optical images showing the transition from microdroplets (top) to microspheres (bottom). Reproduced with permission.^[^ ^24^ ^]^ Copyright 2025, Elsevier. B) Photopolymerization of a poly(anhydride) network and subsequent photodegradation in a dye aqueous solution using catalyst‐loaded microcapsules under UV light exposure (40 min). Reproduced with permission.^[^ ^160^ ^]^ Copyright 2020, Royal Society of Chemistry. C) Sketch of the solvent exchange method to form surface microdroplets followed by local photopolymerization to fabricate microlenses (ML). Reproduced with permission.^[^ ^39^ ^]^ Copyright 2024, Wiley. D) Optical images of random PMMA ML (MLR) and microlens arrays (MLA) on homogeneous and prepatterned substrates.^[^ ^38^ ^]^ Copyright 2023, Elsevier. E) Simulated cross‐sectional light intensity profiles of single ML from PMMA MLR (left) and MLA (right).^[^ ^23^ ^]^ Copyright 2022, American Chemical Society. F) Correlation between photodegradation efficiency (η) and ML focusing effect strength (I_Total_).^[^ ^173^ ^]^ Copyright 2024, Wiley. G) Schematic of a surface ML‐functionalized curved substrate using a flower‐shaped reactor as an example.^[^ ^173^ ^]^ Copyright 2024, Wiley. H) MLA‐functionalized reactor for photocatalytic degradation tested in real river water under varying solar intensities.^[^ ^38^ ^]^ Copyright 2023, Elsevier. I) Sketch of a non‐contact‐mode reactor integrated with a concave MLA PDMS film, including a photo of the film in natural light and SEM image of the surface. J) Simulated cross‐sectional (top) and top‐view (bottom) intensity profiles of concave MLA.^[^ ^39^ ^]^ Copyright 2024, Wiley.

This synthetic strategy has progressively shifted from bulk emulsification to microfluidic systems, which allow superior control over droplet uniformity, internal architecture, and interfacial reactivity. For instance, Li et al.^[^ ^24^ ^]^ employed a capillary microfluidic device to fabricate triethylamine‐functionalized polystyrene/methyl methacrylate microspheres through UV‐induced free radical polymerization. The resulting porous particles exhibited finely tuned surface chemistry, enabling high nitrate removal efficiencies of up to 46.59 mg g^−1^ (**Figure** **7**A). Ho et al.^[^ ^159^ ^]^ leveraged a triple‐emulsion microfluidic approach to engineer asymmetric polyethylene glycol diacrylate microparticles embedded with glucose oxidase and catalase. Their dimpled morphology, formed at the microdroplet interface, endowed the particles with self‐propelling behavior, enabling the collection and elimination of over 75% of micro/nanoplastics from water. Similarly, Liu et al.^[^ ^160^ ^]^ utilized a water‐in‐oil‐in‐water capillary microfluidic system to encapsulate photocatalysts within hydrogel shells, producing microcapsules that enabled highly efficient photocatalytic oxidation of organic dyes and improved catalyst recyclability (Figure 7B). Recent review articles have comprehensively examined the design and synthesis of such functional materials using microfluidic systems.^[^ ^161^, ^162^, ^163^ ^]^

In contrast to freely moving microdroplets in emulsions or those travelling within microfluidic channels, surface microdroplets formed directly on solid substrates are essential when spatial control over product deposition is required. These microreactors offer high stability, precise spatial confinement, and compatibility with in situ imaging and post‐processing, which make them ideal for engineering customizable 2D or 3D material architectures. Zhang and collaborators^[^ ^164^ ^]^ established a solvent exchange method for producing surface nanodroplets, initially adapted from techniques developed for creating surface nanobubbles.^[^ ^165^, ^166^, ^167^ ^]^ This solvent exchange process involves injecting a good solvent containing the solute into a poor solvent, inducing solute supersaturation and triggering femtoliter‐scale droplet formation.^[^ ^168^, ^169^, ^170^ ^]^ A follow‐up study^[^ ^169^ ^]^ demonstrated a quantitative correlation between the Peclet number during solvent exchange and the final droplet size.

One of the earliest applications of surface microdroplets generated via solvent exchange involved the formation of photopolymerizable monomer droplets on hydrophobic substrates. Upon UV irradiation, these droplets were converted into polymeric microlenses (Figure 7C).^[^ ^22^, ^171^ ^]^ These microlenses retained the well‐defined geometric characteristics of the initial droplets, with a predictable ≈30% isotropic size reduction. This shrinkage could be finely tuned by adjusting parameters such as solution composition, flow rate, and chamber geometry. The use of these microlenses for water treatment applications is discussed in Section 4.2.2. Since then, the solvent exchange technique has been extended to include multicomponent liquids and reactive droplet systems, significantly broadening its utility for immobilized microstructured material synthesis.^[^ ^1^, ^172^ ^]^

#### 4.2.2 From Surface‐Bound Reactive Microdroplets to Microlenses for Solar‐Driven Water Decontamination

Surface‐bound functional materials are essential for advancing water and wastewater treatment technologies, particularly in processes requiring energy efficiency, scalability, and long‐term stability.^[^ ^174^, ^175^, ^176^, ^177^ ^]^ By optimizing interactions at the substrate‐contaminant interface, these materials improve performance in adsorption, catalysis, and membrane‐based filtration. Engineered surfaces can enhance pollutant selectivity for recovery and simplify downstream processing.^[^ ^175^ ^]^ Similarly, modified membranes and catalytic surfaces offer high efficiency with reduced energy input in separation and advanced oxidation processes (AOPs).^[^ ^178^, ^179^, ^180^, ^181^, ^182^, ^183^, ^184^ ^]^ Among these, optically active materials stand out due to their ability to effectively harness and redistribute solar energy, making them specially valuable for sustainable water treatment strategies. Their integration into treatment systems allows for enhanced light‐driven processes, such as evaporation, pollutant degradation, and disinfection, under natural sunlight or UV exposure.^[^ ^185^, ^186^ ^]^

Surface microlenses fabricated via the solvent exchange method from reactive microdroplets exhibit strong light‐focusing properties and can be directly immobilized onto treatment surfaces.^[^ ^187^ ^]^ This configuration enables localized optical field enhancement for photodegradation of organic contaminants in wastewater.^[^ ^187^, ^188^, ^189^, ^190^, ^191^ ^]^ To optimize the performance of photodegradation reactors utilizing surface microlenses, their geometric parameters and spatial arrangement are crucial, as these factors determine the resulting light profile after redistribution. Compared to other microlens fabrication techniques such as laser lithography,^[^ ^192^ ^]^ hot embossing,^[^ ^190^ ^]^ or 3D printing,^[^ ^193^ ^]^ the surface microdroplet method offers precise tunability of droplet curvature and spacing by simply modifying surface wettability, providing an energy‐efficient route to control microlens geometry and layout.

On homogeneous hydrophobic surfaces (e.g., silanized glass or Si wafer), the solvent exchange process generates polydisperse random microlenses (MLR), while patterned surfaces yield highly ordered microlens arrays (MLA) (Figure 7D). Both MLR and MLA configurations improve solar‐driven photodegradation of organic contaminants.^[^ ^23^ ^]^ Compared to MLR, MLA exhibits a more pronounced optical focusing effect due to a higher contact angle resulting from the constant contact radius growth mode of microdroplets confined in circular patterns. The shorter focal lengths and the greater light intensity at the focal points, confirmed through optical simulations (Figure 7E), can lead to significantly faster degradation,^[^ ^23^ ^]^ which is particularly advantageous for planar photodegradation reactors that require consistent and intensified optical fields.

Key microlens parameters, including curvature and focal length, can be readily tuned during the solvent exchange process by adjusting variables such as the solution flow rate, chamber height, and the concentration of droplet liquid in the precursor solutions. A focusing intensity index (I_Total_) has been introduced to quantify optical enhancement in an area, defined as the sum of focal intensities of microlenses over a surface area of 1 cm^2^.^[^ ^23^ ^]^ For MLR with I_Total_ exceeding ≈2.3 W cm^−2^, the photodegradation efficiency (η) initially increased rapidly, followed by a slower rate as *I*_Total_ continued to rise (Figure 7F). Overall, MLA achieved a higher photodegradation efficiency than some MLR with greater *I*_Total_, a result attributed to the uniform focal alignment of MLA structures, which permits enhanced concentrations of reactive species within a confined reaction zone.

Surface microlenses fabricated from microdroplets present a promising approach to address critical limitations in the development of photoreactors with enhanced light utilization efficiency: scalability, applicability, and durability. The solvent exchange method facilitates scalable microlenses fabrication by enabling their in situ formation directly on the inner surfaces of glass reactors, including those with complex or non‐planar geometries. This design flexibility allows for the creation of novel reactor configurations that optimize surface‐to‐volume ratios, a key parameter for improving solar energy harvesting. A notable demonstration of this potential involved a flower‐shaped reactor functionalized with surface microlenses (Figure 7G), which exhibited a 12‐fold increase in photodegradation efficiency. Additionally, the method has been applied to functionalize surfaces as large as 257 cm^2^ with microlenses, achieving more than an 80‐fold enhancement in the degradation of methyl orange under simulated sunlight.^[^ ^173^ ^]^

Beyond scalability, surface microlenses also enhance the versatility of photoreactor applications. They are compatible with a broad range of photodegradation processes, including catalytic systems. For example, reactors integrated with MLA showed a clear improvement in ZnO‐catalyzed degradation of four different micropollutants under standard sunlight conditions.^[^ ^38^ ^]^ The enhancement factor was observed to increase at reduced irradiance levels, reaching ≈30% higher at 0.3 Sun compared to 1 Sun. The adaptability of MLA was further confirmed in real‐world scenarios: in river water matrices (Figure 7H), where light transmission was hindered by dissolved organic matter and particulates, MLA integration still improved photocatalytic degradation efficiency by 25%.^[^ ^194^, ^195^, ^196^, ^197^ ^]^ This demonstrates the robustness of surface microlenses and their capacity to maintain performance under challenging conditions for light‐driven AOP.

To address long‐term operational stability,^[^ ^198^ ^]^ a reusable PDMS film embedded with concave MLA was fabricated using a soft lithography method.^[^ ^199^, ^200^ ^]^ In this process, convex MLA structures generated via solvent exchange served as molds to imprint inverse geometries into optically clear and flexible PDMS films. SEM imaging (Figure 7I) confirmed high‐fidelity replication of lens dimensions, and the transparency and flexibility of PDMS facilitated seamless integration into various reactor configurations. Confocal microscopy and optical simulations (Figure 7J) revealed that the concave MLA focused incident light into hot spots located 9–17 µm beneath the PDMS surface. A non‐contact configuration ‐ where the PDMS‐MLA film is suspended above the aqueous phase ‐ prevents direct interaction with the liquid, reducing the risk of lens detachment or leaching. This design achieved a 6‐fold enhancement in methyl orange degradation under weak illumination (0.4 Sun),^[^ ^39^ ^]^ showcasing the potential of PDMS‐based MLA as durable, reusable, and contamination‐free photonic structures for solar‐driven water treatment.

#### 4.2.3 From Surface‐Bounded Reactive Microdroplets to Metal Oxide Photocatalyst

Surface‐bound metal oxide catalysts play a pivotal role in AOPs for water and wastewater treatment due to their ability to generate ROS that can degrade persistent organic contaminants.^[^ ^201^ ^]^ However, their implementation is often hindered by post‐synthesis challenges associated with conventional fabrication methods, particularly difficulties in material recovery, separation, and long‐term recyclability.^[^ ^202^ ^]^ In this context, microdroplet‐based synthesis presents a promising and scalable alternative, enabling streamlined fabrication and deposition processes, reduced reagent consumption, and finely tunable reaction environments for tailoring material properties. Metal oxide materials synthesized from microdroplets can serve two functions: as active catalytic agents and as support matrices that stabilize catalytic species.

Kuai et al.^[^ ^27^ ^]^ developed a micro‐gas‐blasting technique to generate microdroplets in spray form, thereby facilitating the scalable synthesis of single‐atom metal oxide. Within each droplet, the rapid decomposition of nitrates and combustion of glucose produced explosive gas evolution, which facilitated the formation of ultrathin CeO_2_ nanosheets and uniform dispersion of single Pt atoms. The resulting Pt_1_/CeO_2_ catalysts demonstrated over a 2‐fold increase in catalytic activity compared to their microspherical analogues, while maintaining robust atomic dispersion and structural stability. In another study, Zhang and colleagues^[^ ^96^ ^]^ reported a fast and continuous microdroplet‐confined assembly method to fabricate Cu(0)/Cu(I)@TiO_2_ microsphere photocatalyst. The precursor solution ‐ comprising titanium (IV) tetraisopropoxide and Cu(NO_3_)_2_ ‐ was aerosolized through a nozzle, and microdroplets were rapidly dried and calcinated. During this process, capillary forces at the microdroplet‐gas interface facilitated the formation of compact microspheres. The intimate interface between Cu and anatase enhanced electron transfer and copper redox cycling. Under solar irradiation and in the presence of peroxymonosulfate, the catalyst achieved a 5‐fold increase in pollutant degradation within just 10 min.

**Figure 8:**
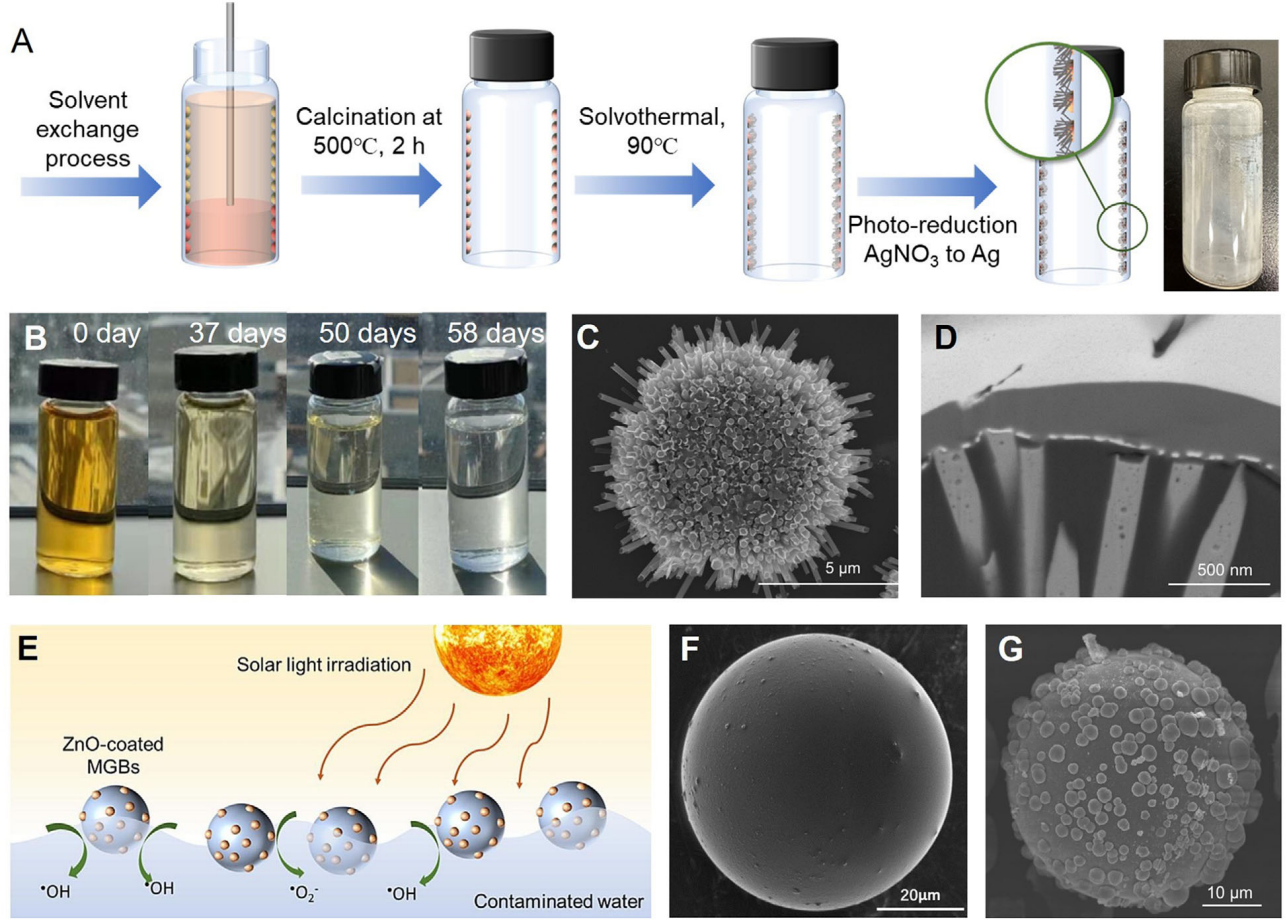
A) Fabrication procedure and photograph of ZnO nanocaps‐functionalized vials using solvent exchange and hydrothermal treatment. Reproduced with permission.^[^ ^40^ ^]^ Copyright 2025, Elsevier. B) Color change of methyl orange solution in a ZnO nanocaps‐functionalized vial under simulated indoor sunlight. Reproduced with permission.^[^ ^26^ ^]^ Copyright 2023, American Chemical Society. C) SEM (top view) and D) PFIB‐SEM (cross‐sectional view) images of ZnO nanorods. Reproduced with permission.^[^ ^40^ ^]^ Copyright 2025, Elsevier. E) Schematic illustration of ZnO‐coated hollow micro glass bubbles, with FESEM images of F) uncoated MGB and G) ZnO‐coated MGB. Reproduced with permission.^[^ ^203^ ^]^ Copyright 2024, Elsevier.

Surface‐bound metal oxide materials synthesized from microdroplets can directly serve as photocatalyst within reactors. This approach eliminates the need for post‐use catalyst recovery and improves reusability in AOP for wastewater treatment. Wei and collaborators^[^ ^25^ ^]^ and Li et al.^[^ ^26^ ^]^ introduced a synthesis pathway based on the solvent exchange process to form metal oxide nanocaps from nanodroplets containing organometallic precursors. As displayed in **Figure** **8**A, oleic acid microdroplets formed on hydrophobic surfaces served as soft templates. Upon reaction with zinc acetate, zinc oleate formed in situ, which was subsequently calcinated at 500 °C to yield ZnO nanocaps. These ZnO‐functionalized substrates significantly improved the photocatalytic degradation of methyl orange (Figure 8B), as well as of pharmaceutical pollutants such as norfloxacin and sulfamethoxazole, by 3.2‐fold and 7.2‐fold, respectively. However, the photocatalytic activity was limited by the total mass of immobilized ZnO.

A surface nanodroplet‐assisted method was developed using ZnO nanocaps as seeds for the anisotropic formation of ZnO nanorods.^[^ ^40^ ^]^ These nanocaps served as heterogeneous nucleation sites, enabling radial alignment and anisotropic extension of ZnO nanocaps to form porous and circular clusters (Figure 8C). Focused ion beam (FIB) and cross‐sectional SEM imaging revealed a rough and multilayered structure (Figure 8D). Growth‐time studied indicated that while rod length slightly increased, its diameter significantly expanded, suggesting a lateral growth‐dominant mechanism. The resulting high‐porosity ZnO nanorods served as the effective scaffold for hybrid photocatalyst fabrication.

Photocatalytic efficiency of ZnO nanorods was highly dependent on their growth time, which influenced both length and diameter. Optimal performance was achieved through a balance between increased surface area (from longer nanorods) and sufficient inter‐rod spacing (controlled by diameter). Upon Ag functionalization, the Ag/ZnO nanorods exhibited markedly enhanced degradation efficiency across a mixture of eight persistent micropollutants in water. This enhancement was attributed to plasmonic effects, improved charge separation, and extended light absorption. The most significant improvement was observed for atrazine (ATZ), with a 3.06‐fold increase in degradation efficiency, while other pollutants showed enhancements ranging from 1.33‐ to 2.15‐fold relative to unmodified ZnO nanorods. Importantly, Ag/ZnO‐coated reactors demonstrated excellent reusability and material stability, maintaining nearly 100% degradation efficiency over four consecutive treatment cycles without evidence of photocorrosion or metal leaching.^[^ ^40^ ^]^ This robustness highlights the strong potential of immobilized Ag/ZnO nanostructures for reliable, scalable, and safe application in solar‐driven water treatment systems.

Suspension‐based photocatalysts often exhibit suboptimal light absorption due to limited light penetration in water, where only ≈20% of visible light and less than 1% of UV light can reach a depth of 50 cm.^[^ ^204^, ^205^ ^]^ To overcome this limitation, a novel strategy involves incorporating photocatalysts onto floatable substrates, which improves light harvesting by maintaining the catalyst at the water surface while also facilitating easy post‐treatment separation (Figure 8E).^[^ ^206^, ^207^, ^208^ ^]^ Hollow micro glass bubbles (MGB) are particularly well‐suited as floating supports due to their low density, spherical geometry, thermal stability, and chemical inertness.^[^ ^203^ ^]^ ZnO‐coated micro‐glass bubbles (ZnO‐MGB) were evaluated for photocatalytic degradation of a mixture of seven micropollutants under dark and simulated solar irradiation. While bare MGB (Figure 8F) showed negligible adsorption or photolysis activity, ZnO‐MGB (Figure 8G) achieved rapid and efficient degradation for all micropollutants, including ATZ, one of the most recalcitrant compounds in the mixture. The ZnO‐MGB retained high photocatalytic efficiency over three consecutive reuse cycles, with only a slight decline observed in the fourth, likely attributable to material loss during recovery. Throughout the tests, ZnO‐MGB remained morphologically intact, underscoring the robustness, stability, and reusability of the microdroplet‐assisted ZnO‐MGB system for advanced solar‐driven water treatment applications.

#### 4.2.4 Metallic Materials from Reactive Microdroplets

**Figure 9:**
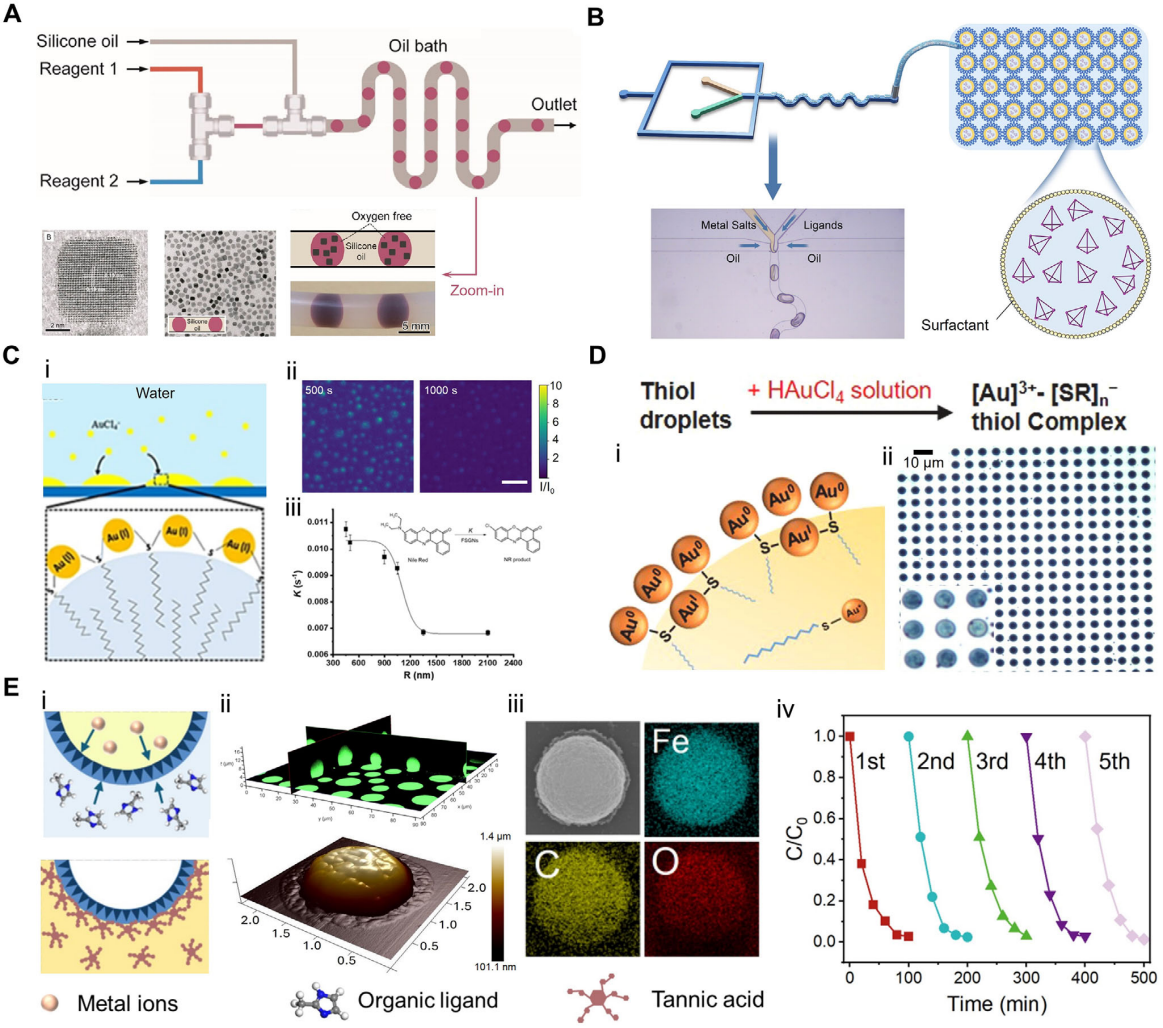
A) A sketch of the microfluidic device for the synthesis of Pt nanoparticles, with silicone oil as the carrier phase. TEM and HRTEM images of Pt cubes were displayed. Reproduced with permission.^[^ ^209^ ^]^ Copyright 2022, American Chemical Society. B) Schematic of the microdroplet generated with a microfluidic device for metallacage preparation.^[^ ^210^ ^]^ Copyright 2023, Wiley. C) (i) Schematic of the biphasic reaction at surface nanodroplets interface to form flower‐shaped surface gold nanostructures; (ii) Confocal microscopy images of the binary surface nanodroplets at 500 s and 1000 s; (iii) Rate of catalytic reaction of the gold nanostructures as a function of the droplet radius. Reproduced with permission.^[^ ^211^ ^]^ Copyright 2024, Wiley. D) (i) Reaction schematic of HAuCl_4_ precursor interaction with the 1‐dodecanthiol surface nanodroplets; (ii) Optical images of the array of gold nanocraters (GNCs). Reproduced with permission.^[^ ^212^ ^]^ Copyright 2022, Elsevier. E) (i) Formation of MOF at the liquid–liquid interface of surface microdroplets; (ii) 3D‐stacked confocal fluorescence microscopy image of surface microdroplets dyed with FITC‐Dextran (up) and AFM image of a single MIL‐100 dome (down); (iii) SEM‐EDX images of a single MIL‐100 dome; (iv) Reusability tests of the MIL‐100 substrate for model compounds removal in water. Reproduced with permission.^[^ ^31^ ^]^ Copyright 2025, Elsevier.

Surface nano‐/microdroplets can also be used as miniaturized reactors for synthesizing metal‐based functional materials, offering valuable opportunities for catalytic applications in water treatment. For example, Niu et al.^[^ ^209^ ^]^ developed a silicon oil droplet‐based synthesis method that achieved an 81% yield of cubic Pt nanoparticles with a high production rate (31.8 mg·min^−1^) and outstanding catalytic performance in methanol oxidation (**Figure** **9**A). Beyond improved kinetics, microdroplets provide distinct compartmentalized environments that shield sensitive reactions from atmospheric oxygen or moisture,^[^ ^135^, ^209^ ^]^ while enabling fine‐tuned control over precursor mixing and nucleation. Lin and collaborators ^[^ ^210^ ^]^ demonstrated this using a microfluidic system in which internal recirculation within aqueous droplets ensured thorough mixing of metal salts and organic ligands (Figure 9B), rapidly yielding well‐defined metallacages in just minutes. The metallacages exhibited catalytic activities more than 2.22 times higher than those produced in conventional batch systems, underscoring promising performance in versatile catalytic reactions.

Surface microdroplets formed via solvent exchange can reach a number density ranging from several million to over 100 million per cm^2^ in a single batch experiment. Yu et al.^[^ ^211^ ^]^ exploited this process to fabricate flower‐shaped surface gold (Au) nanostructures with complex 3D morphologies using binary surface nanodroplets composed of dodecanethiol and octanol (Figure 9C). Upon formation of these droplets on a substrate, HAuCl_4_ was introduced into the aqueous phase. An interfacial reaction between ${\mathrm{AuCl}}_{4}^{-}$ ions and the dodecanethiol‐rich microdroplets led to the formation of [Au(I)–SC12H25] complexes (Figure 9C‐i), resulting in the localized growth of highly branched Au nanostructures. These surface‐confined Au assemblies exhibited higher surface areas compared to conventional Au nanoparticles and demonstrated enhanced catalytic activity for the degradation of Nile red dye (Figure 9C‐ii,iii). In a related approach, Dabodiya et al.^[^ ^212^ ^]^ utilized surface‐bound thiol microdroplets obtained as spatially confined reaction sites for the sequential deposition of Au ions within a microfluidic flow chamber (Figure 9D‐i). This strategy produced well‐ordered arrays of surface‐anchored Au nanocraters with a diameter of 4 µm (Figure 9D‐ii). The nanocraters served as catalytic hot spots, exhibiting efficient degradation performance for both anionic and cationic azo dyes in aqueous media. This approach provides a separation‐free and scalable route to fabricating catalytic surfaces, offering significant advantages for large‐area water purification technologies.

#### 4.2.5 Metal–Organic Framework (MOF) Functional Materials

MOF have attracted significant attention for water remediation applications due to their exceptionally high porosity, large surface area, abundant active sites, and tunable chemical functionalities.^[^ ^213^, ^214^ ^]^ While traditional MOF synthesis techniques often require harsh conditions or complex post‐treatment, microdroplet‐based methods provide compelling advantages. These include rapid crystallization, continuous processing capability, controlled particle size and morphology, and reduced reliance on toxic solvents.^[^ ^72^, ^215^ ^]^ Within the confined environments of microdroplets, rapid mixing and reaction rates promote fast nucleation and growth of MOF crystals with well‐defined structures and enhanced properties. For instance, aerosol generated by ultrasonic spray or electrospray has enabled the synthesis of hollow materials or dense MOF spheres such as HKUST‐1 and ZIF‐8 at the liquid–air interface, where solvent evaporation drives nanocrystal assembly.^[^ ^216^, ^217^, ^218^ ^]^ Alternatively, liquid–liquid interface assembly in emulsion or microfluidic systems offers excellent control over encapsulation and interfacial MOF growth, producing materials suited for multifunctional water treatment.^[^ ^72^, ^219^, ^220^ ^]^ However, many of these techniques yield MOF suspended in bulk environments, complicating their collection and limiting their integration into spatially structured systems for practical remediation applications.

Wu et al.^[^ ^31^ ^]^ developed a microfluidic strategy for fabricating surface‐immobilized MOF with tunable morphology and positioning. In the process, water microdroplets were immobilized on (3‐Aminopropyl)‐ triethoxysilane (APTES)‐coated glass slides through solvent exchange in a laminar flow reactor. A ternary solvent mixture (octanol, ethanol, and water; solution A) was displaced by water‐saturated octanol (solution B), generating sessile aqueous droplets (Figure 9E‐i). Due to selective partitioning, metal precursors preferentially accumulated in the aqueous phase, while subsequently introduced organic linkers (solution C) diffused in from the organic phase, initiating MOF crystallization at the droplet interface. This interfacial reaction yielded dome‐shaped MOF structures, as confirmed by atomic force microscopy (AFM) and D‐stacked fluorescence imaging (Figure 9E‐ii), with characteristic wrinkled textures indicative of MIL‐100 formation. The method proved to be highly versatile, enabling the synthesis of additional MOF‐HKUST‐1, MIL‐88A, and ZIF‐8 on both rigid and flexible substrates (e.g., PDMS). Morphology, distribution, and crystallinity were readily tuned via flow control and reactor geometry. Optimized MIL‐100 domes (Figure 9 (E‐iii)) showed over a 5‐fold enhancement in photocatalytic degradation of organic pollutants via Fenton‐like mechanisms and retained performance across five treatment cycles (Figure 9 (E‐iv)).

## 5 Reactive Microdroplets for Clean Energy Production

The confined environments created by microdroplets have opened new avenues for clean energy production, enabling reactions that are otherwise inefficient or challenging under bulk conditions. These microscale reactors provide a versatile platform for carrying out catalytic transformations central to sustainable energy technologies. For example, they have been employed to facilitate the transesterification of triglycerides for biodiesel production and the dehydrogenation of organosilanes for on‐demand hydrogen release. This section begins by highlighting recent developments in the use of Pickering emulsion microdroplets to perform these two representative processes. While comprehensive reviews exist on Pickering emulsions and microgel‐ or surfactant‐stabilized droplets in general,^[^ ^221^, ^222^, ^223^, ^224^ ^]^ we focus here on their targeted application to energy‐relevant catalysis.

We then turn to air–liquid interfaces of water microdroplets where ROS are spontaneously generated. These ROS‐rich interfaces have recently been utilized to drive chemical reactions related to nitrogen fixation and the synthesis of value‐added nitrogen‐containing compounds. In contrast to stabilized emulsions, these systems rely on droplet generation methods that produce transient yet highly reactive aqueous interfaces without the use of surfactants or nanoparticles. The discussion begins with catalytic approaches using water microdroplets and subsequently explores emerging catalyst‐free strategies for nitrogen conversion. Finally, we explore the growing use of liquid metal microdroplets as catalytic platforms in emerging energy‐related processes.

### 5.1 Pickering Emulsion Microdroplets in Catalytic Biodiesel Synthesis

**Figure 10:**
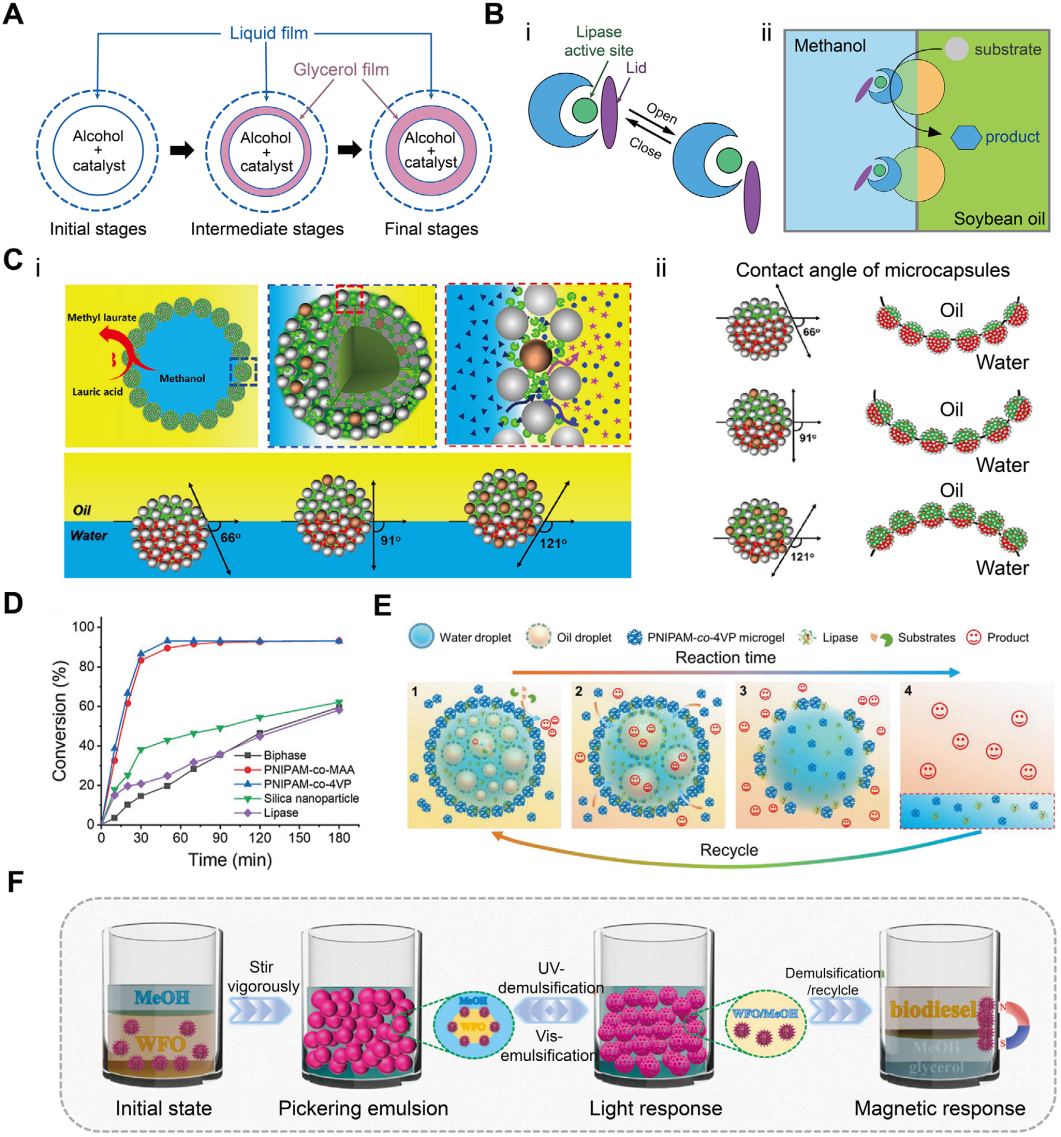
A) Schematic illustration showing the film zone where the transesterification reaction occurs and the subsequent formation of a glycerol layer at the interface. Reproduced with permission.^[^ ^232^ ^]^ Copyright 2019, Elsevier Ltd. B) Mechanism of the enzymatic transesterification of soybean oil: (i) the closed (inactive) and open (active) conformations of the enzyme structure, and (ii) schematic of enzymatic interfacial activation. Reproduced with permission.^[^ ^237^ ^]^ Copyright 2024, Elsevier B.V. C) Schematic highlighting the interfacial localization of microcapsule systems with varied wettability. Reproduced with permission.^[^ ^238^ ^]^ Copyright 2022, American Chemical Society. D) Catalytic conversion of substrates in different biphasic systems. E) Schematic representation of interfacial biocatalysis and lipase recycling using O/W/O Pickering double emulsions co‐stabilized by PNIPAM‐*co*‐4VP microgels and lipase. Reproduced with permission.^[^ ^239^ ^]^ Copyright 2022, John Wiley and Sons. F) Magneto‐optical responsive behavior of the Pickering emulsion. Reproduced with permission.^[^ ^240^ ^]^ Copyright 2024, Elsevier.

The growing demand for renewable and environmentally friendly fuels has intensified efforts to identify alternatives to conventional petroleum‐based diesel. Combustion of traditional diesel is associated with significant emissions of particulate matter and nitrogen oxides, which contribute to air pollution and climate change.^[^ ^225^, ^226^ ^]^ Biodiesel has emerged as a promising substitute, a renewable fuel derived from biological feedstocks such as vegetable oils or animal fats.^[^ ^227^ ^]^ It consists of fatty acid alkyl esters (FAAE), typically produced via transesterification of triglycerides with short‐chain alcohols in the presence of homogeneous, heterogeneous, or enzymatic catalysts.^[^ ^228^, ^229^ ^]^ Compared to fossil diesel, biodiesel offers several environmental advantages, including carbon neutrality, enhanced biodegradability, and lower emissions of harmful pollutants.^[^ ^230^, ^231^ ^]^ However, the transesterification reaction is limited by the immiscibility between the oil and alcohol phases, which creates substantial mass transfer resistance. Studies such as those by Chanakaewsomboo and colleagues^[^ ^232^, ^233^ ^]^ demonstrated that the reaction takes place at the oil‐alcohol interface, specifically within a thin triglyceride film zone (**Figure** **10**A). Consequently, the available interfacial area becomes a critical parameter governing conversion efficiency.

Pickering emulsion microdroplets have been employed as an effective strategy to enhance the interfacial area between immiscible oil and alcohol phases.^[^ ^234^, ^235^, ^236^ ^]^ For instance, Zhang et al.^[^ ^237^ ^]^ developed a Pickering emulsion stabilized by a Janus interfacial catalyst containing lipase to catalyze the conversion of soybean oil into biodiesel (Figure 10B). Under optimized conditions, the Pickering interfacial biocatalyst achieved a biodiesel yield of 87.33%, higher than the 66.51% yield obtained using a free enzyme system. In a more complex design, Zeng and colleagues^[^ ^238^ ^]^ engineered a lipase‐entrapped colloidosome (LEC) system that integrates biocatalysts with a mixture of hydrophilic and hydrophobic SiO_2_ nanoparticles (Figure 10C). This hybrid assembly enabled the formation of stable Pickering emulsions specifically tailored for the esterification of lauric acid with methanol. The resulting biodiesel yield ranged from 69.97% to 80.37%, compared to only 9.82% achieved with free lipase under biphasic conditions. This enhanced performance is attributed to the strategic localization of LEC at the oil‐water interface, which increased the available reaction area and improved mass transfer efficiency between the immiscible reactants and catalytic sites. Moreover, the confined interfacial microenvironment created by the microcapsules likely protects the enzyme structure and promotes the stability of the catalytic system under dynamic reaction conditions.

Several studies have investigated the integration of polymers into Pickering emulsions to improve emulsion stability. A notable example is the work by Meir et al.,^[^ ^241^ ^]^ who investigated lipase activity within double‐shell cellulose‐coated oil‐in‐water emulsions. These Pickering emulsions exhibited enzymatic activity ranging from 38 to 76%, in stark contrast to the negligible 1% activity of free lipase dispersed in water. The novelty of this approach lies in its ability to facilitate a transesterification pathway rather than hydrolysis,^[^ ^242^ ^]^ even in an aqueous environment containing more than 90 wt.% water. Poly(*N*‐isopropylacrylamide) (PNIPAM)‐based microgels have also been employed as stabilizers in Pickering double emulsions for biodiesel production.^[^ ^239^ ^]^ When combined with lipase, these microgel‐stabilized systems (PNIPAM‐*co*‐MAA and PNIPAM‐*co*‐4VP) achieved a 90% conversion efficiency, compared to only 20% conversion in biphasic system with free lipase in solution (Figure 10D). This enhanced catalytic performance stems from the ability of microgels to form responsive, semi‐permeable interfacial barriers that concentrate substrates and enzymes at the oil‐water interface. Furthermore, the spontaneous phase separation that occurs upon substrate depletion allows for easy product recovery, as the emulsion breaks down after the reaction (Figure 10E).

Despite the significant advantages of Pickering emulsions in promoting transesterification reactions, a persistent challenge lies in achieving rapid and reversible emulsification/demulsification switching.^[^ ^243^ ^]^ To address this limitation, Guo et al.^[^ ^240^ ^]^ developed magneto‐optical polymeric nanospheres functionalized with Brønsted–Lewis di‐acid ionic liquids. These multifunctional nanospheres not only reduced mass transfer resistance across alcohol/oil biphasic systems but also enabled dynamic control over emulsification and demulsification through UV irradiation (Figure 10F). In a related effort, Li and co‐workers^[^ ^244^ ^]^ utilized light‐responsive Janus nanoparticles to catalyze biodiesel synthesis with reversible switching between emulsified and de‐emulsified states triggered by alternating UV and visible light. Under optimized conditions, the system achieved a biodiesel yield of 97.9%. These photoresponsive Pickering emulsions demonstrate a promising strategy for integrating high‐yield transesterification with catalyst recyclability, advancing the sustainability and efficiency of microdroplet‐assisted chemical synthesis.

### 5.2 Hydrogen Production from Microdroplets of Liquid Organic Hydrogen Carriers

**Figure 11:**
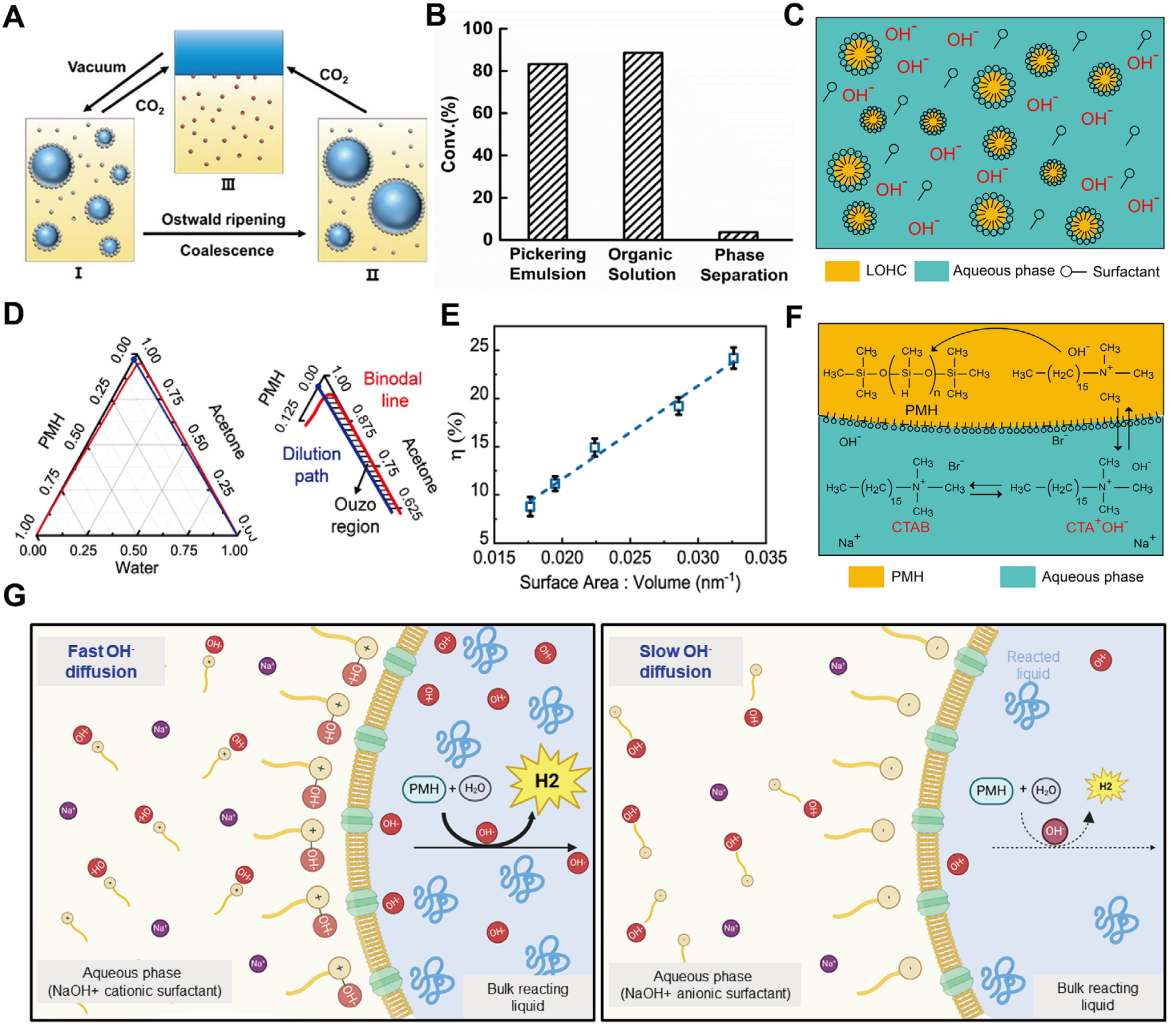
A) Schematic representation of the programmable reaction process via CO_2_/vacuum treatment.Reproduced with permission.^[^ ^249^ ^]^ Copyright 2019, American Chemical Society. B) Histogram of organosilane conversion efficiency in different systems: Pickering emulsion, the presence of organic solvent, and two‐phase system. Reproduced with permission.^[^ ^249^ ^]^ Copyright 2019, American Chemical Society. C) Schematic of dispersed organosilane microdroplets in an aqueous environment. Reproduced with permission.^[^ ^53^ ^]^ Copyright 2025, American Chemical Society. D) Ternary phase diagram of the PMH‐acetone‐water system used to prepare surfactant‐free Ouzo emulsions. Reproduced with permission.^[^ ^54^ ^]^ Copyright 2025, Elsevier Inc. E) Effect of surface‐to‐volume ratio on H_2_ conversion yield (η). Reproduced with permission.^[^ ^54^ ^]^ Copyright 2025, Elsevier Inc. F) Schematic of OH^−^ ion transfer from the aqueous to the organic phase mediated by CTAB.Reproduced with permission.^[^ ^53^ ^]^ Copyright 2025, American Chemical Society. G) Influence of surfactant type on the interfacial hydrogen evolution reaction: (left) cationic surfactants enable fast hydroxide ion diffusion; (right) anionic and non‐ionic surfactants hinder diffusion. Reproduced with permission.^[^ ^53^ ^]^ Copyright 2025, American Chemical Society.

Hydrogen is widely recognized as a clean energy carrier due to its high energy density and carbon‐free combustion. However, its practical deployment is limited by challenges in storage and transport, such as low volumetric density and the need for high‐pressure or cryogenic containment systems.^[^ ^245^, ^246^ ^]^ To overcome these limitations, hydrogen storage in the form of liquid organic carriers has gained interest. Among these, organosilane derivatives have attracted attention for their high hydrogen content, chemical stability, and low toxicity.^[^ ^247^, ^248^ ^]^ Despite these advantages, the dehydrogenation of organosilanes in aqueous environments to release H_2_ is also often hindered by the immiscibility between the organic and aqueous phases, which lead to sluggish reaction kinetics. In 2019, Zhang and co‐workers^[^ ^249^ ^]^ introduced a microdroplet‐based strategy by investigating the oxidation of dimethylphenylsilane (Me_2_PhSiH) Pickering emulsion stabilized by a CO_2_‐responsive microgel (**Figure** **11**A). The emulsion enabled the formation of microdroplets with a total interfacial area of ≈0.23 m^2^ using only 2 mL of Me_2_PhSiH, an increase of nearly three orders of magnitude compared to conventional biphasic systems. As a result, the Pickering emulsion achieved a conversion efficiency of 83% and a turnover frequency of 166.4 h^−1^, in contrast to the two‐phase system, which showed only 4% conversion and a turnover frequency of 7.4 h^−1^ (Figure 11B). However, a gradual decline in catalytic activity was observed over time, attributed to a barrier effect imposed by the microgel shell, which limited mass transport and reactant contact. The authors overcame this challenge by employing cyclic CO_2_ injection and vacuuming, which triggered microgel collapse and re‐swelling, restoring interfacial accessibility and sustaining reaction performance.

Surfactants have also been employed to stabilize organosilane emulsion microdroplets during dehydrogenation reactions. Our group recently investigated this strategy using emulsions of polymethylhydrosiloxane (PMH) dispersed in a basic aqueous environment.^[^ ^53^ ^]^ Regardless of the specific surfactant employed, dispersing PMH as microdroplets (Figure 11C) proved to be more efficient for H_2_ generation than the conventional two‐phase system. Moreover, the rate of H_2_ generation increased with decreasing droplet size, due to the corresponding rise in total interfacial area. A linear relationship was observed between the amount of H_2_ released and the total interfacial area. A similar trend was obtained using surfactant‐free emulsions formed via the Ouzo effect (Figure 11D,E).^[^ ^54^ ^]^ Beyond merely stabilizing the emulsions, surfactants were found to influence reaction kinetics. Anionic (sodium dodecyl sulfate) and non‐ionic (Tween 20) surfactants adsorb at the interface and form interfacial layers that hinder the diffusion of OH^−^ ions (Figure 11F).^[^ ^250^ ^]^ In contrast, CTAB (cetyltrimethylammonium bromide) and other quaternary ammonium surfactants act as phase‐transfer catalysts and enhance H_2_ formation rate by facilitating the transfer of OH^−^ ions into the organic phase (Figure 11G). Such inhibitory and promoting effects of surfactants on interfacial reaction kinetics have been reported in various systems.^[^ ^251^, ^252^, ^253^ ^]^ However, this study is the first to demonstrate surfactant‐mediated control of interfacial hydrogen evolution with microdroplet‐based catalytic systems.

**Figure 12:**
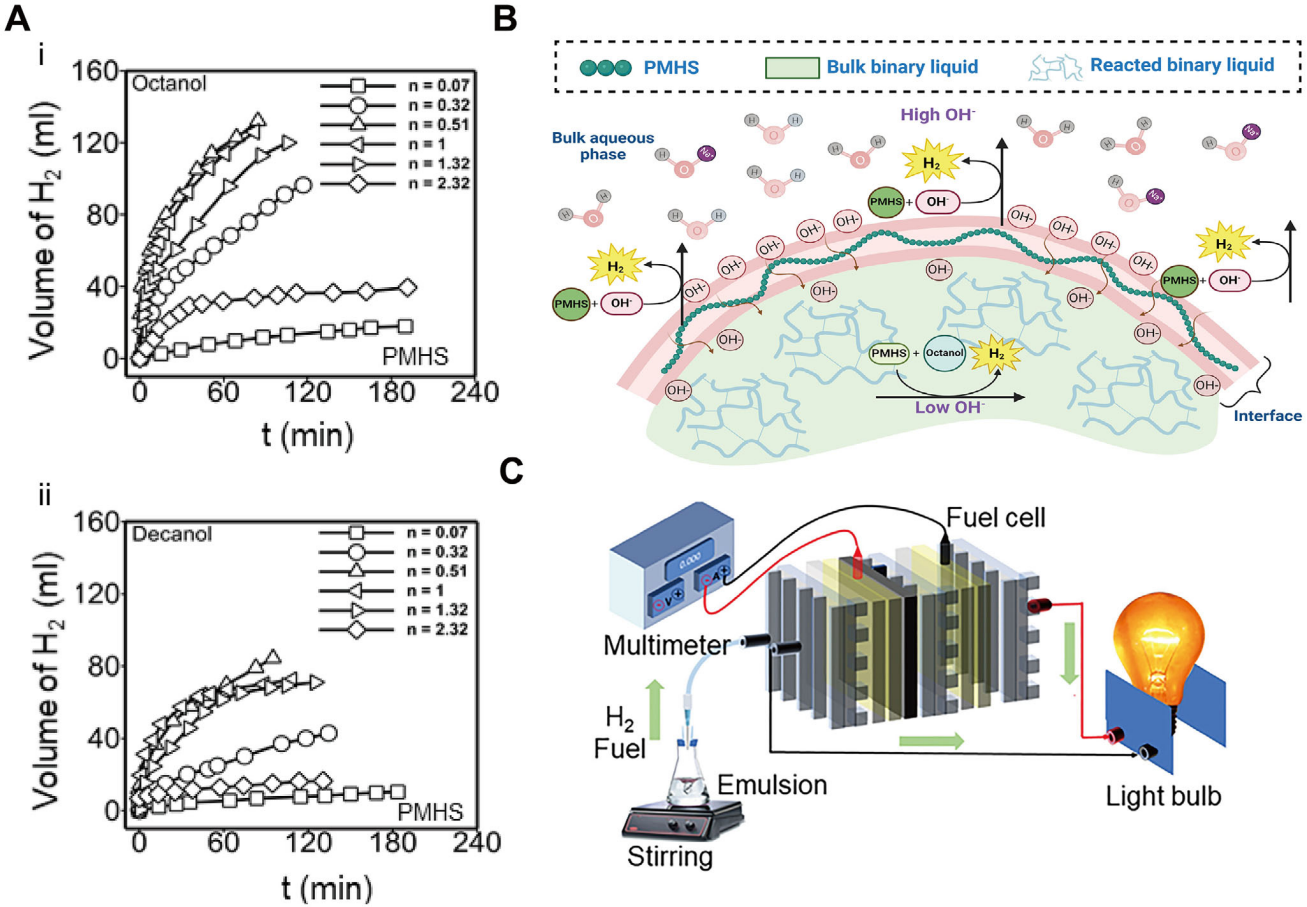
A) Time evolution of H_2_ volume at varying mixing factors (*n*) for binary (i) PMH‐octanol and (ii) PMH‐decanol microdroplets. Reproduced with permission.^[^ ^254^ ^]^ Copyright 2025, Elsevier B.V. B) Schematic of a single organosilane droplet reacting in a surrounding sodium hydroxide solution, where dehydrogenation proceeds within the droplet via alcoholysis and at the droplet‐water interface via hydrolysis. Reproduced with permission.^[^ ^254^ ^]^ Copyright 2025, Elsevier B.V. C) Schematic of an energy conversion system utilizing a single‐unit fuel cell powered by H_2_ generated from a bulk reaction. Reproduced with permission.^[^ ^254^ ^]^ Copyright 2025, Elsevier B.V.

An attractive alternative to nanoparticle‐ and surfactant‐stabilized systems was recently introduced by Dogra and colleagues^[^ ^254^ ^]^ who demonstrated fast H_2_ release from binary microdroplets without the use of any stabilizing agents. It was found that mixing organosilanes with a long‐chain alcohol, such as decanol or octanol, leads to more efficient H_2_ generation (**Figure** **12**A,B). H_2_ production gradually increased with the mixing ratio (*n*) because of the dual reaction sites enabled by the binary microdroplet configuration. Hydrolysis occurs at the interface between organosilanes and water, while a secondary alcoholysis reaction takes place within the droplet between the alcohol and organosilanes (Figure 12C). However, the reaction rate declined beyond a certain mixing factor. A similar trend was reported by Voronova et al.^[^ ^255^ ^]^ who observed a decrease in reaction rate when the alcohol concentration exceeded a threshold. This was attributed to the potential self‐association of alcohols, forming (HOR)_*x*_ or (RO^−^)(HOR)_*x*_ clusters that hinder the interaction between organosilanes and alcohol molecules. A systematic analysis involving three organosilanes revealed that the maximum H_2_ production occurred at *n* = 1. To demonstrate the practical applicability of droplet‐based systems, the reaction setup was connected to a fuel cell, where the generated H_2_ was converted into electricity to power a small light bulb (Figure 12D). Mitsudome and colleagues^[^ ^256^ ^]^ have also developed a catalytic system for on/off switchable H_2_ production from organosilanes, contributing to the development of next‐generation green hydrogen fuel cells with on‐demand H_2_ generation.

### 5.3 Nitrogen Fixation on Water Microdroplet Surfaces

**Figure 13:**
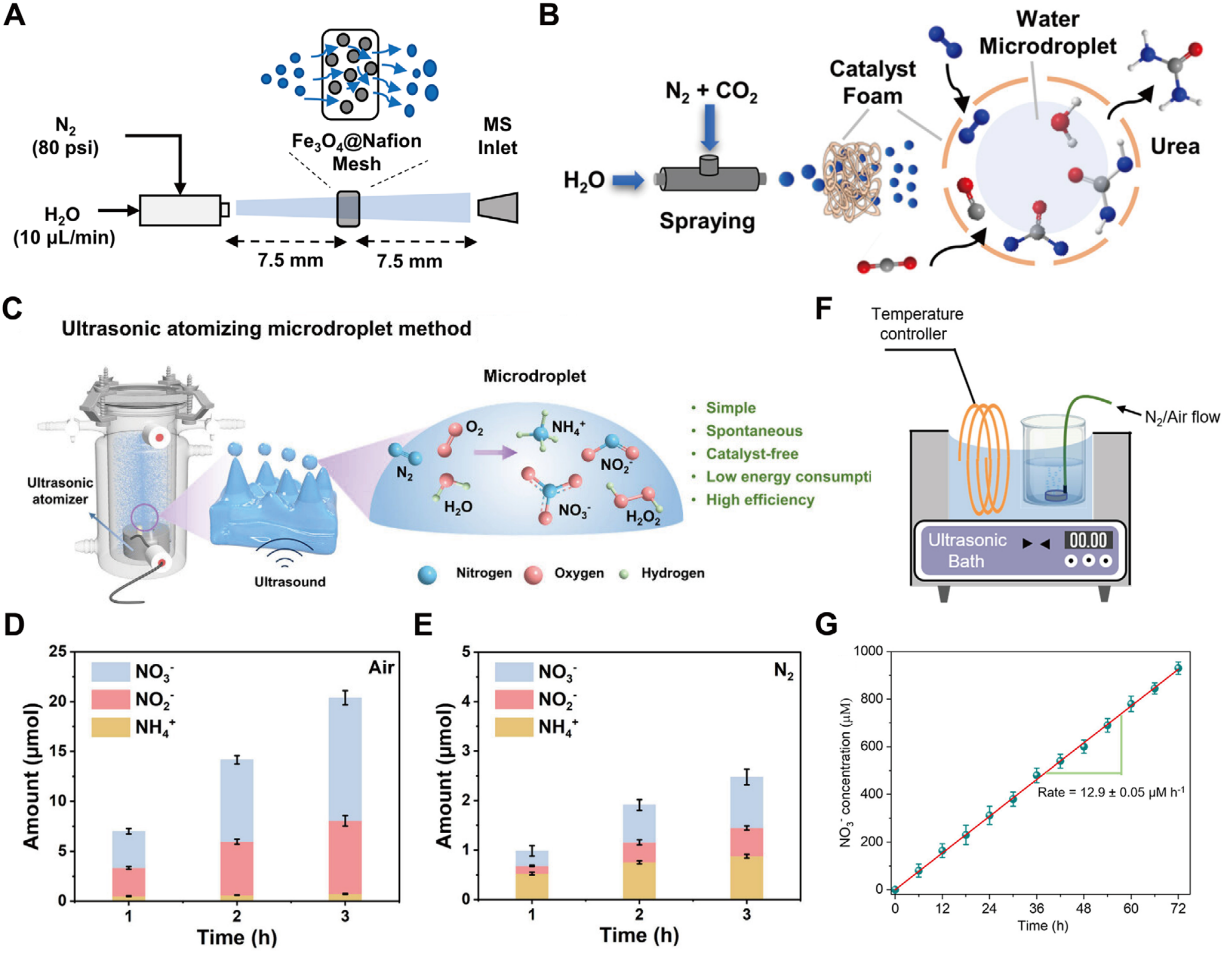
A) Ultrasonic spray setup coupled with mass spectrometry for real‐time monitoring of ammonia formation. Reproduced with permission.^[^ ^50^ ^]^ Copyright 2023, Proceedings of the National Academy of Sciences. B) Schematic of the microdroplet‐based system for urea synthesis from CO_2_ and N_2_.^[^ ^261^ ^]^ C) Schematic of the ultrasonic atomization method for nitrogen fixation. Time‐dependent evolution of nitrogen fixation products under D) air and E) N_2_ atmospheres. Reproduced with permission.^[^ ^51^ ^]^ Copyright 2025, American Chemical Society. F) Experimental setup for HNO_3_ production. G) ${\mathrm{NO}}_{3}^{-}$ yield as a function of N_2_ bubbling time. Reproduced with permission.^[^ ^262^ ^]^ Copyright 2024, American Chemical Society.

We now turn to the most recent and timely application of microdroplet reaction: nitrogen fixation. This process plays a key role in clean energy technologies by enabling the sustainable production of fuels and hydrogen carriers. In particular, compounds such as ammonia (NH_3_), derived from nitrogen fixation, show great potential as high‐capacity hydrogen storage materials and alternative fuels.^[^ ^257^, ^258^, ^259^ ^]^ Recent studies have revealed that the air‐liquid interfaces of water microdroplets offer unique reaction environments that can drive nitrogen fixation processes.^[^ ^260^ ^]^ For example, Song and co‐workers^[^ ^50^ ^]^ employed magnetic iron oxide (Fe_3_SO_4_) as a catalyst to synthesize NH_3_ on the surface of water microdroplets, using either air or N_2_ as the nebulizing gas. This system enabled NH_3_ production within just 0.2 ms, achieving a conversion rate of up to 32.9 ± 1.38 nmol·s^−1^·cm^−2^. Using a catalyst mesh composed of Fe_3_SO_4_ embedded in a Nafion polymer (**Figure** **13**A), they further reported NH_3_ concentrations as high as 270.2 ± 25.1 µM within 2 h.^[^ ^49^ ^]^ The same group also demonstrated single‐step urea synthesis on the sub‐millisecond timescale (Figure 13B).^[^ ^261^ ^]^ Despite these promising results achieved at room temperature without applied voltage or light, the presence of active catalysts remained indispensable.

More recently, Wang et al.^[^ ^51^ ^]^ proposed a catalyst‐free nitrogen fixation strategy under ambient conditions, utilizing ultrasonically atomized water microdroplets in contact with air or pure N_2_ within a custom‐designed reactor (Figure 13C). In this system, a variety of reactive nitrogen species formed at the dynamic air‐water interface, ultimately yielding ${\mathrm{NH}}_{4}^{+}$, ${\mathrm{NO}}_{2}^{-}$, and ${\mathrm{NO}}_{3}^{-}$ as final products. After 1 h, oxidative nitrogen species dominated when air was used as the gas source, resulting in a total fixed nitrogen yield of 6.99 µmol, which is higher than the 0.97 µmol obtained in a pure N_2_ environment (Figure 13D,E). The reported nitrogen fixation rates per hour ranged from 3.5 to 220 times higher than those achieved by alternative methods under similar conditions. Gas‐water interfaces of microbubbles have also been explored for the spontaneous formation of H_2_O_2_ and its downstream role in driving nitric acid synthesis. A representative study by Bose et al.^[^ ^262^ ^]^ demonstrated a linear increase in ${\mathrm{NO}}_{3}^{-}$ concentration over a period of 132 h, achieving an average production rate of 12.9 ± 0.05 µM h^−1^. Remarkably, this redox‐driven nitrogen fixation occurred without the need for external voltage or light irradiation (Figure 13F). These findings highlight the potential of air‐water interfacial systems to drive redox reactions relevant to sustainable nitrogen fixation, even in the absence of traditional energy inputs.

Researchers have proposed that ROS spontaneously generated at the interfaces of microdroplets and microbubbles can activate N_2_ or even cleave the strong N ≡ N triple bond, enabling catalyst‐free nitrogen fixation under ambient conditions.^[^ ^51^, ^262^, ^263^, ^264^ ^]^ As mentioned before, similar reactive radicals can also be produced using cold plasma technology, which facilitates the formation of nitrogen oxides (NO_*x*_) and their anionic counterparts (${\mathrm{NO}}_{x}^{-}$).^[^ ^265^, ^266^ ^]^ When coupled with suitable downstream processes, these reactive intermediates can be efficiently converted into ${\mathrm{NO}}_{3}^{-}$ through thermodynamically favorable pathways.^[^ ^267^, ^268^ ^]^ A recent review from our group offers a comprehensive overview of these emerging interfacial and plasma‐assisted nitrogen fixation strategies.^[^ ^269^ ^]^

### 5.4 Emerging Microdroplet Strategies for Catalysis

Beyond their established role as reactants for the generation of clean energy carriers, microdroplets are increasingly being investigated for a broader range of catalytic applications in sustainable energy technologies. Recent developments include microdroplets derived from deep eutectic solvents, which offer a green and tunable medium for catalytic processes due to their low volatility, ionic character, and capacity to stabilize reactive intermediates.^[^ ^270^, ^271^ ^]^ Similarly, microdroplets formed from high‐entropy alloys present exciting possibilities for catalytic diversification, enabling adjustable surface compositions and synergistic active sites for complex reactions.^[^ ^272^, ^273^ ^]^

Among these emerging directions, liquid metal microdroplets have received particularly significant attention, either as catalysts or as platforms for fabricating catalytic systems targeting key chemical processes such as CO_2_ reduction and hydrocarbon dehydrogenation. Their fluidic nature offers distinct advantages over conventional solid‐state catalysts: they eliminate grain boundary defects, facilitate continuous surface renewal, and enhance mass transport at the reaction interface.^[^ ^274^ ^]^ In addition, their exceptional electrical conductivity supports efficient interfacial electron transfer, while their strong resistance to surface deactivation ensures sustained catalytic performance.^[^ ^275^ ^]^

Kalantar–Zadeh's research group has made substantial contributions to the development of liquid metal microdroplets, advancing both the mechanistic understanding and practical deployment of these systems for energy‐related catalytic processes.^[^ ^52^, ^280^, ^283^, ^287^, ^289^, ^290^ ^]^ **Table** **1** highlights key studies from this group and others. Among the various processes explored, the reduction of CO_2_ into solid carbonaceous materials is one of the most thoroughly investigated directions.^[^ ^274^ ^]^ In a recent patent (US‐20230219068‐A1), Tang and colleagues^[^ ^291^ ^]^ introduced a strategy that uses dispersed liquid metal microdroplets as catalysts or catalytic systems for CO_2_ reduction. Their approach involves mechanically dispersing liquid metal or alloy into an immiscible solvent to generate catalytically active microdroplets. These microdroplets facilitate the direct chemical reduction of CO_2_ at the droplet interface, forming carbon‐carbon bonds without the need for an external electrical input. In a complementary system, Ye et al.^[^ ^276^, ^277^ ^]^ dissolved metals such as magnesium (Mg) and lithium (Li) into liquid gallium (Ga) to create room‐temperature alloys capable of converting CO_2_ into solid carbon. During the reaction, Mg/Li atoms migrate toward the gas–liquid interface, where they reduce CO_2_ while undergoing oxidation. Notably, this electrochemical mechanism supports a closed‐loop process, as the oxidized species can be regenerated through reduction.

**Table 1:** Summary of liquid metal and alloy microdroplets in energy‐relevant catalytic processes.

Reaction type	Liquid metals	Products	Refs.
CO_2_ reduction	Ga‐Sn‐Ni	O_2_, C_*s*_ ^a^	[52]
	Ga‐Mg		[276]
	Ga‐Li		[277]
	Ga‐Sn/In	HCOO^−^	[278]
	Ga‐Bi		[279]
Hydrocarbon dehydrogenation	Ga‐Ni	H_2_, C_*n*_H_2*n*_ ^b^	[280]
	Ga‐Pt		[281]
	Ga		[282]
	Ga‐Sn‐Ni		[283]
	Ga‐Pt		[284]
	Ga‐Pt		[285]
	Ga‐Pd		[286]
	Ga	H_2_, CH_4_	[287]
	Ga‐Ni	H_2_, CO	[288]

^a^ *C*_s_: carbonaceous materials (C═C and C─O bonds).

^b^ C_*n*_H_2*n*_: Alkenes.

The Ga‐containing metal approach has also proven to be effective in converting CO_2_ to formate (HCOO^−^). For instance, Liu et al.^[^ ^278^ ^]^ successfully performed this electrochemical conversion using Ga–Sn and Ga–In alloys. The formate Faradaic efficiency reached up to 98% with liquid‐phase alloys, compared to a maximum of 30% observed for their solid‐state counterparts. While Sn or In atoms in solid alloys aggregated into phase‐segregated clusters, they remained atomically dispersed within the Ga matrix in the liquid state. This atomic dispersion significantly increased the local electron density around the active sites, thereby enhancing the catalytic selectivity for formate. Moreover, the inherent atom mobility and redox reversibility of the liquid phase introduced a self‐healing capability in the Ga–Sn and Ga–In systems, which suppressed surface oxidation and maintained catalytic performance. These self‐healing properties were further explored by Hou et al.,^[^ ^279^ ^]^ who used a Ga–Bi alloy under extended operation. After observing electrocatalytic degradation over time, they demonstrated that simple mechanical agitation followed by in situ regeneration could fully restore catalytic activity. These studies underscore the critical relationship between structural dynamics, electronic states, and catalytic behavior in liquid metal alloy droplets.

Liquid metal alloy microdroplets have also been employed to catalyze the dehydrogenation of hydrocarbons into alkenes and H_2_. This reaction is inherently endothermic and requires elevated temperatures, and many conventional liquid‐phase catalytic systems fail. In contrast, the thermal stability and unique properties of liquid metal alloys make them particularly well‐suited for such conditions.^[^ ^292^ ^]^ Tang et al.^[^ ^283^ ^]^ demonstrated the selective production of propylene from decane and canola oil using Ga–Sn–Ni microdroplets at 150°, achieving propylene selectivities of 90.5% and 94.5%, respectively. Notably, by increasing the total droplet surface area from 19.5 to 2,725 cm^2^, they achieved a ≈30‐fold enhancement in catalytic efficiency. Raman et al.^[^ ^284^ ^]^ further demonstrated that liquid metal alloy microdroplets can operate effectively under harsher thermal conditions, specifically in the range of 500–600 °C. The authors successfully performed propane dehydrogenation using Ga‐Pt microdroplets. The catalytic system exhibited a propylene selectivity above 93% at 500 °C. However, at temperatures exceeding 550°, the selectivity declined due to the formation of stable side products that competed with propene formation.

These studies illustrate the dual capability of liquid metal microdroplets to address two fundamental challenges in clean energy technologies: mitigating atmospheric CO_2_ through catalytic transformation and producing value‐added chemical products such as formate, carbonaceous materials, alkenes, and alkanes. Their exceptional interfacial activity, tunable composition, and stability under both ambient and extreme conditions make them a versatile and promising platform for catalytic processes across a range of temperatures and feedstocks. Continued exploration of their structural dynamics and regeneration mechanisms will be essential for translating this emerging technology into scalable real‐world applications.

## 6 Chemically‐Powered Microdroplet Motion

Microdroplets are traditionally considered as confined reaction vessels; however, when rendered mobile through internal or interfacial reactions, they can act as self‐propelled microengines capable of performing complex functions in liquid environments. Such dynamic behavior has garnered increasing interest, particularly for applications in environmental remediation. Recent studies have demonstrated that self‐propelled microdroplets and micromotors can actively participate in tasks such as the degradation of organic pollutants,^[^ ^293^, ^294^ ^]^ removal of heavy metals,^[^ ^47^, ^295^ ^]^ extraction of oil contaminants,^[^ ^296^, ^297^ ^]^ and even the destruction of pathogenic bacteria.^[^ ^298^, ^299^, ^300^ ^]^ Unlike static droplets, where molecular transport is dominated by diffusion, self‐propelled droplets induce convective flows as they move through the surrounding medium. This motion facilitates more efficient transport of reactants to the droplet interface and removal of products from it, potentially enhancing interfacial reaction rates. For example, in nanoextraction processes, droplet motion relative to the surrounding fluid improves extraction efficiency by increasing interfacial fluxes, which are governed by partition coefficients and mass transfer kinetics sensitive to convective conditions.

When driven by external forces or Marangoni effects (surface tension gradients), droplet motion can induce complex internal flow patterns that enhance mixing of reactants, thereby accelerating reaction kinetics. In addition, the motion of microdroplets can lead to transient deformations in their shape, resulting in fluctuating surface areas that expose new interfacial sites for reactions. These dynamic interfacial changes help prevent the accumulation of reaction byproducts or inhibitors, which might otherwise hinder catalytic activity. The combined effects of enhanced mass transport, increased interfacial area, and continuous exposure of fresh reactive surfaces contribute to more uniform and sustained reaction rates. Thus, droplet motion is not merely a passive consequence of the system but an active driver of reactivity, modulating chemical reactions through its influence on convective transport, collision frequency, and interfacial dynamics.

For the rational design and optimization of droplet‐based material synthesis and chemical analysis platforms, a thorough understanding of microdroplet motion is essential. Significant research efforts have focused on uncovering the physicochemical mechanisms underlying microdroplet mobility, including propulsion direction, velocity, and lifetime. Typically, their self‐propulsion arises from interfacial tension gradients induced by chemical reactions, micelle‐induced solubilization, or phase transitions.^[^ ^301^ ^]^ A comprehensive review of the physicochemical hydrodynamics of such active droplet systems and their relevance for practical applications has been provided by Lohse and colleagues.^[^ ^1^ ^]^ In this final section, we briefly summarize key developments in the study of chemically driven autonomous microdroplet motion.

**Figure 14:**
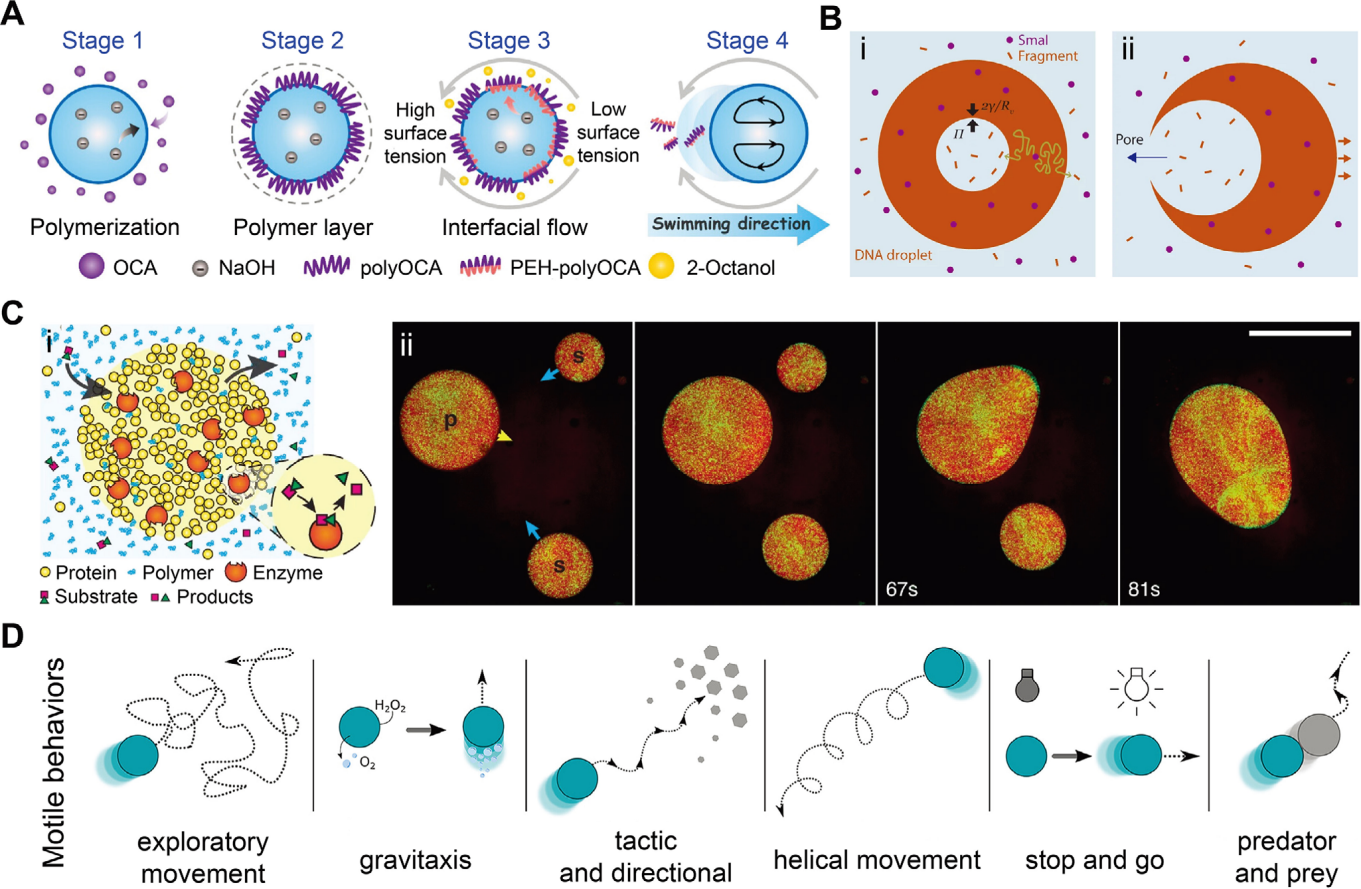
A) Schematic representation of polymer‐based self‐swimming microdroplet. Reproduced with permission.^[^ ^302^ ^]^ Copyright 2025, American Chemical Society. B) Mechanistic interpretation of vacuole growth rates and popping‐based motility. Reproduced with permission.^[^ ^303^ ^]^ C) (i) Schematic representation of a chemically active protein condensate. Polymers act as depletants, triggering condensation, while enzyme‐rich droplets function as micro‐chemical reactors. (ii) Image sequence of chemically active droplets on a non‐wetting surface, showing the primary droplet (p), satellite droplets (s), and the direction of motion indicated in the first panel. Reproduced with permission.^[^ ^304^ ^]^ D) Schematic illustration of different motile behaviors involving biomolecules. Reproduced with permission.^[^ ^305^ ^]^ Copyright 2023, Elsevier Inc.

Chemical reactions within microdroplets can act as internal driving forces for autonomous motion by generating surface tension gradients. These gradients typically arise from the localized consumption of reactants, accumulation of products, or reaction‐induced changes triggered by external stimuli such as light. A notable example was demonstrated by Lian et al.^[^ ^302^ ^]^, who developed polymer‐based self‐swimming microdroplets. In their system, the motion was achieved through the synergistic effects of rapid interfacial polymerization and hydrolysis reactions (**Figure** **14**A), which produced a weak amphiphilic polymer. The accumulation of this polymer at the droplet interface created a surface tension gradient. This gradient, in turn, induced strong Marangoni circulation flows along the oil‐water interface, effectively propelling the microdroplets.

Biomolecule‐containing microdroplets have also been developed to achieve controlled propulsion and environmental responsiveness. A notable example is provided by Saleh et al.^[^ ^303^ ^]^ who demonstrated that internal structural formation can drive directional motion of DNA microdroplets. Enzyme activity generated vacuole‐like compartments within the droplets, which gradually expanded and eventually bursted at the interface (Figure 14B). The collapse of these vacuoles produced an osmotic imbalance that propelled the droplet forward through a burst‐like motion driven by internal pressure. In another example, Jambon‐Puillet and collaborators^[^ ^304^ ^]^ investigated active droplets composed of urease‐loaded bovine serum albumin, which exhibited internal flows driving collective mobility (Figure 14C). Smaller satellite droplets were observed to accelerate as they approached a larger primary droplet, which itself attracted satellites over farther distances. These long‐range interactions were attributed to concentration gradients in solutes consumed and/or produced by the enzymatic reaction. Beyond these mechanisms, other types of motility have been reported in biological systems, including exploratory movement, gravitaxis and ballistic propulsion, tactic and directional behaviors, helical movement, and stop‐and‐go dynamics (Figure 14D).^[^ ^305^ ^]^

**Figure 15:**
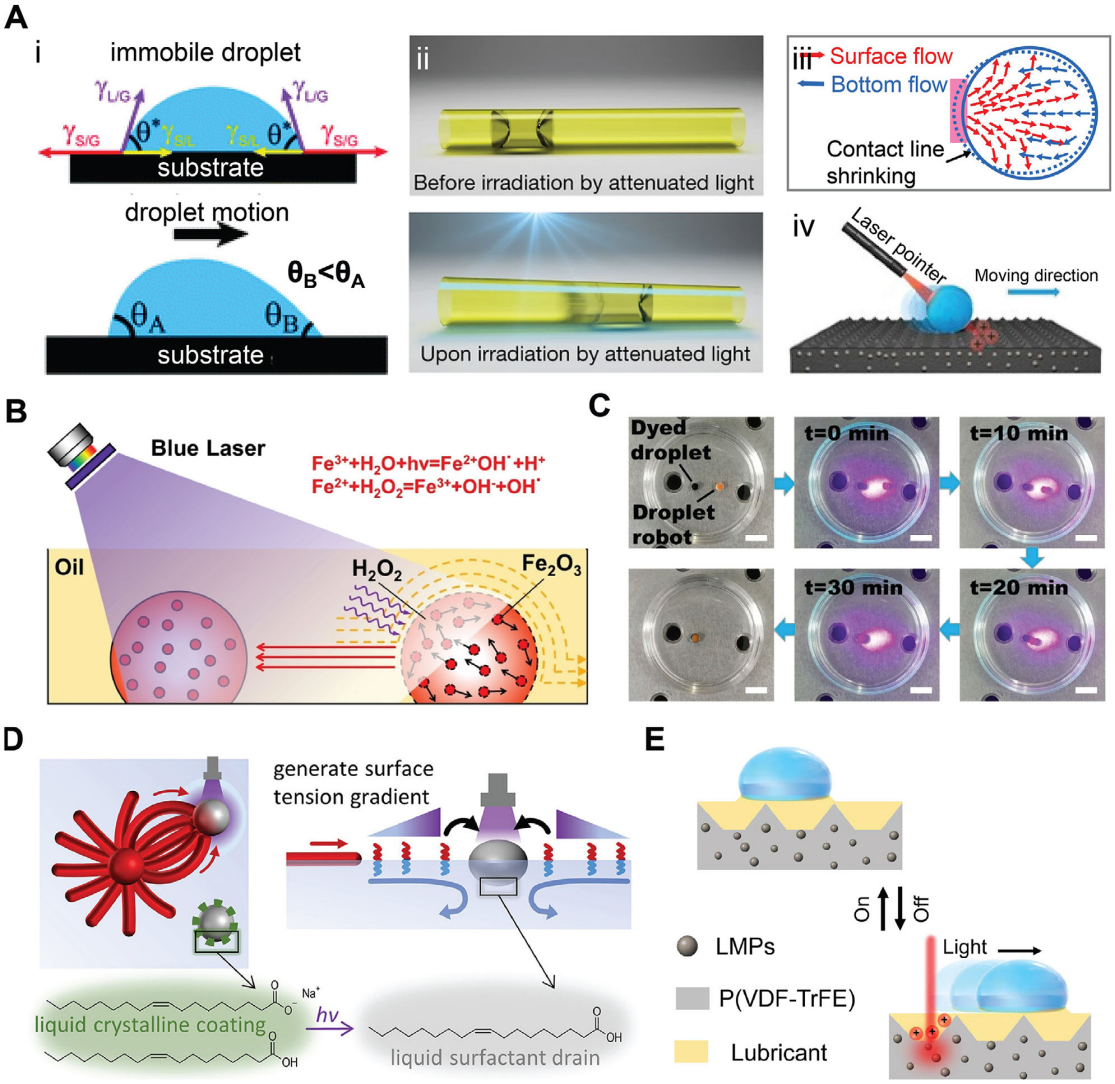
A) Schematic representations of various mechanisms underlying light‐induced droplet motion: (i) A wettability gradient induces a difference in contact angles between the rear and front of the droplet, generating a Laplace pressure gradient and internal flow. Reproduced with permission.^[^ ^306^ ^]^ Copyright 2012, Royal Society of Chemistry. (ii) Photodeformation‐driven motion of a fully wetting liquid slug confined in a tubular microactuator. Reproduced with permission.^[^ ^307^ ^]^ Copyright 2016, Macmillan Publishers Limited. (iii) Trajectories of tracer beads within a water droplet subjected to near‐infrared (NIR) irradiation at one edge. Reproduced with permission.^[^ ^308^ ^]^ Copyright 2018, Wiley–VCH. (iv) Light‐guided droplet manipulation on a photo‐induced charged surface enabling real‐time, in situ generation of free surface charges. Reproduced with permission.^[^ ^309^ ^]^ B,C) Schematic and time‐lapse snapshots of the experimental setup and motion mechanism of a light‐driven water droplet in an oil phase. Scale bar: 5 mm. Reproduced with permission.^[^ ^310^ ^]^ Copyright 2020, Wiley–VCH. D) Schematic illustration of interconnected droplet–filament networks with photocontrolled drain droplets. Reproduced with permission.^[^ ^311^ ^]^ E) Schematic illustration of a light‐induced charged slippery surface (LICS) for droplet manipulation. Reproduced with permission.^[^ ^312^ ^]^

Light is widely recognized as an effective external stimulus for inducing and controlling the motion of swimming microdroplets, primarily through mechanisms such as photochemical reactions, photomechanical deformation, light‐induced Marangoni flows, and optoelectric effects (**Figure** **15**A).^[^ ^313^ ^]^ In many systems, photogenerated reaction products at the droplet interface are key to initiating and sustaining motion. For instance, Sun et al.^[^ ^310^ ^]^ reported one of the most recent demonstrations of phototaxis behavior, where water microdroplets self‐propelled in response to light stimuli using isotropic semiconductor Fe_2_O_3_ nanoparticles (Figure 15B,C). The droplet continuously moved toward the light source at a speed of ≈3.1 µm·s^−1^. Numerical simulations revealed that a photocatalytic Fenton reaction occurs on the illuminated Fe_2_O_3_ nanoparticles, creating an uneven ion concentration that generated a propulsion‐driving gradient.

Later, Nguindjel et al.^[^ ^311^ ^]^ introduced a system combining surfactants, self‐assembly, and photochemistry to enable chemical signal transfer between droplets (Figure 15D). Their strategy involves three main steps: (i) the formation of a liquid crystalline coating on oleic acid/sodium oleate (OA/NaO) droplets upon contact with water, (ii) disintegration of this coating via a photoacid generator that protonates sodium oleate under UV light, and (iii) creation of a surface tension gradient caused by surfactant depletion at the air‐water interface. Finally, Wang and colleagues^[^ ^312^ ^]^ demonstrated that light‐induced charged slippery surface (LICS) can exert photocontrol of droplets with fast speed, long distance, antigravity motion, and directionally collective motion (Figure 15E). For a broader overview of recent progress in light‐driven droplet mobility, readers are referred to several comprehensive review articles.^[^ ^313^, ^314^, ^315^ ^]^

**Figure 16:**
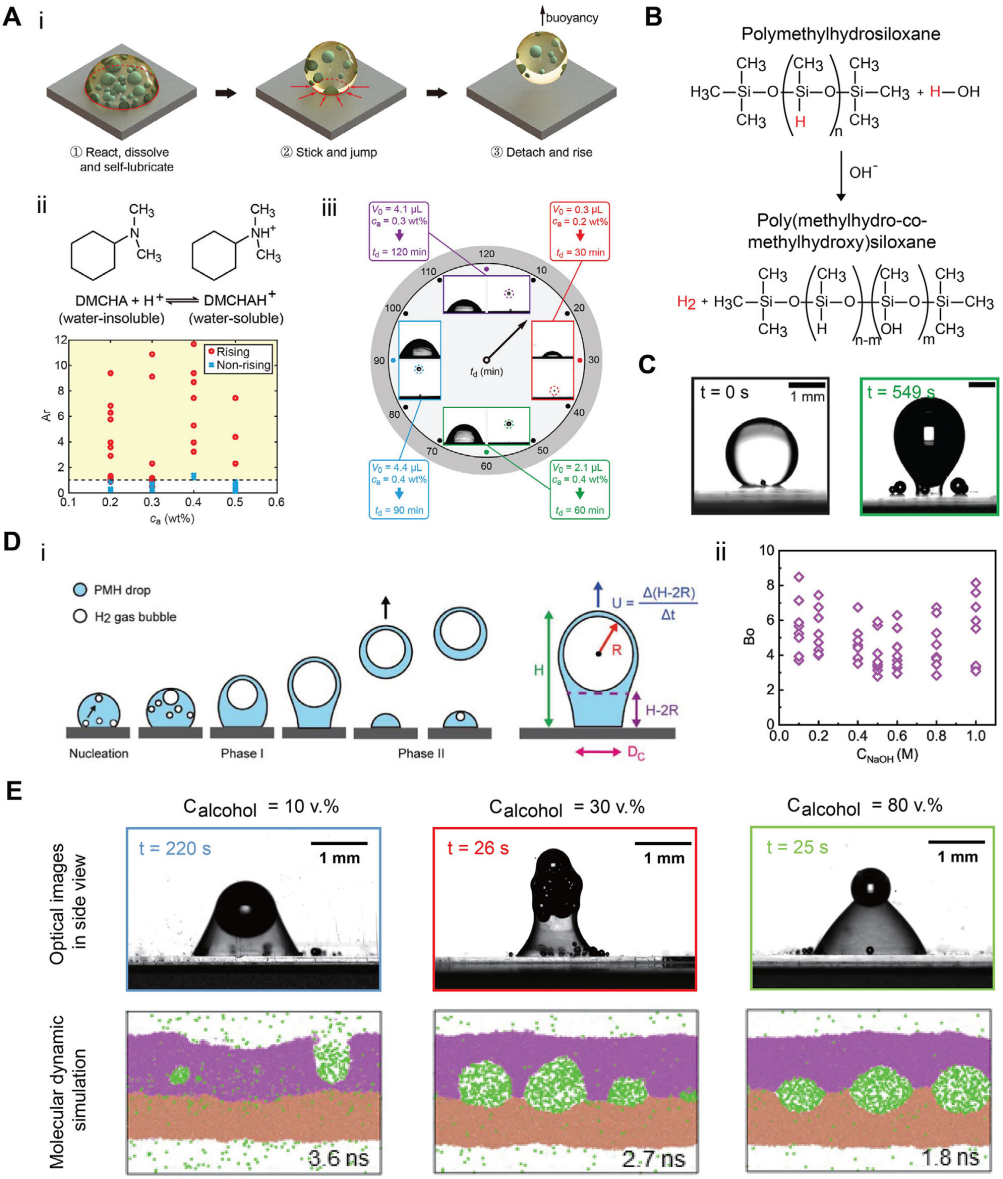
A) (i) Schematic representation of the detachment and rise of a PS‐DMCHA microdroplet from a solid substrate. (ii) Archimedes (Ar) numbers for the non‐rising and rising droplet. (iii) A sketch of setting time for launching the drop with varied initial conditions. Reproduced with permission.^[^ ^316^ ^]^ Copyright 2023, Wiley‐VCH. B) NaOH‐catalyzed dehydrogenation of polymethylhydrosiloxane (PMH) to produce hydrogen gas (H_2_). C) Side view photo and D) (i) Schematic illustration and snapshots of (i) H_2_ bubble nucleation, growth, and in‐drop bubble formation. (ii) Bond numbers for numerous droplets and various NaOH concentrations. Reproduced with permission.^[^ ^317^ ^]^ Copyright 2024, Wiley‐VCH. E) Snapshots showing the growth of H_2_ bubbles from binary PMH–alcohol microdroplets of varying composition. Molecular simulations illustrating nanobubble nucleation and growth at three different interfacial tensions (γ), left: $\gamma_{12}$ = 40 mN m^−1^, middle: $\gamma_{12}$ = 23 mN m^−1^, and right: $\gamma_{12}$ = 10 mN m^−1^. Reproduced with permission.^[^ ^254^ ^]^ Copyright 2025, Elsevier B.V.

Recently, our group identified two distinct mechanisms underlying the spontaneous rise of submerged microdroplets from solid substrates. In the first case, we demonstrated that a binary droplet composed of polystyrene (PS) and a switchable solvent, *N*, *N*‐dimethylcyclohexylamine (DMCHA), gradually shrinks upon the droplets reacting with an acidic solution.^[^ ^316^ ^]^ This induced internal phase separation, leading to the formation of sessile water microdroplets within the host droplet and ultimately causing its detachment from the surface (**Figure** **16**A). In the second case, we reported a novel bubble‐driven rising mechanism involving PMH microdroplets in contact with aqueous NaOH (Figure 16B–D).^[^ ^317^ ^]^ Here, H_2_ bubbles nucleated and grew within the droplet until buoyancy forces lead to detachment. Furthermore, we showed that the location of bubble formation can be precisely modulated using binary PMH‐alcohol microdroplets.^[^ ^254^ ^]^ As the alcohol content increased, the system underwent a regime transition from in‐drop nucleation to clustering, and eventually to on‐drop formation (Figure 16E). Molecular simulations revealed that bubble positioning and contact angle are governed by the balance between surface and interfacial tensions. These findings uncover an unrecognized self‐ejection mechanism and demonstrate that microdroplet motion can be actively tuned through chemical reaction. In particular, when gas is produced in the product and forms bubbles, the rich dynamics of bubble and reactive droplets remains to be further understood, and leveraged for gas production from droplet liquids.

## 7 Conclusion and Outlook

This review has summarized the remarkable versatility of microdroplets as confined reaction environments for the rational design of functional materials targeting critical challenges in water purification and sustainable energy generation. Through a comprehensive analysis of recent literature, we have highlighted how microdroplets significantly enhance chemical sensing and analysis by enabling nanoextraction, facilitating reactive detection schemes, and supporting the fabrication of SERS‐active substrates. Their utility extends to the synthesis of advanced materials for pollutant detection and water remediation, including advanced polymeric optical materials, surface‐bound catalytic nanomaterials, metal oxides, and MOF. In the domain of clean energy, we have underscored the potential of reactive microdroplets in biodiesel synthesis, hydrogen evolution, and the fixation of nitrogen and CO_2_.

Beyond the approaches covered in this review, there is also exciting progress in the development of microdroplet‐based synthetic cell platforms, aiming to mimic key features and behaviors of living cells, deepen our fundamental understanding of cellular processes, and advanced biotechnology and medical applications.^[^ ^318^, ^319^ ^]^ These platforms leverage the unique compartmentalization, dynamic interfacial properties, and tunable chemical environments of microdroplets to recreate essential cellular functions such as signal transduction,^[^ ^320^, ^321^ ^]^ molecular exchange,^[^ ^322^, ^323^ ^]^ enzymatic cascades,^[^ ^324^ ^]^ and even collective behavior.^[^ ^325^, ^326^ ^]^ By serving as simplified yet controllable analogs of living cells, microdroplet‐based synthetic systems provide powerful tools for investigating the physicochemical basis of life‐like behavior and for engineering programmable artificial cells. These advances are helping to bridge materials science and synthetic biology, with promising implications for applications in biosensing,^[^ ^327^, ^328^ ^]^ targeted drug delivery,^[^ ^329^, ^330^ ^]^ and smart biomedical devices.^[^ ^331^ ^]^

Looking ahead, recent developments also point toward the integration of microdroplet systems with artificial intelligence as a transformative frontier for both mechanistic understanding and application development. Machine learning‐assisted molecular dynamics simulations have significantly improved both accuracy and computational efficiency in modeling interfacial molecular processes.^[^ ^332^ ^]^ For instance, the flexible artificial neural network successfully fit a higher‐dimensional reactive potential to ab initio training data, permitting the simulation of reactive uptake of N_2_O_5_ into water droplets.^[^ ^333^ ^]^ Yet, unlike bulk systems, capturing and interpreting interfacial phenomena in reactive microdroplets remains a grand challenge and a rich area for future computational studies.

In parallel, artificial intelligence is accelerating material discovery by enabling autonomous experimentation in microdroplet‐based platforms. A striking example is the modular self‐driven droplet reactor by Volk et al.,^[^ ^334^ ^]^ which integrates reinforcement learning to guide the multi‐step synthesis of semiconductor nanoparticles. Similarly, AI‐enhanced microdroplets are opening new possibilities for real‐time sensing in physiological, environmental, and biomedical contexts. Recent advances in plasmonic wearable devices and sweat sensors exemplify how combining microdroplet‐based sensing with machine learning algorithms can enable rapid data analysis, predictive diagnostics, and advanced human‐machine interactions.^[^ ^335^, ^336^, ^337^, ^338^, ^339^, ^340^, ^341^ ^]^

Altogether, the convergence of reactive microdroplets and AI‐powered automation is poised to redefine high‐throughput material discovery and process optimization. These dynamic and adaptable systems not only have the potential for water purification and clean energy production, but also promise disruptive innovations across diverse fields, from environmental sensing to personalized healthcare.

## Conflict of Interest

The authors declare no conflict of interest.
